# Dose-dependent gibberellic acid and benzylaminopurine priming coordinates transcriptional and metabolic reprogramming, enhancing photochemical efficiency to confer drought tolerance in pigeonpea

**DOI:** 10.3389/fgene.2026.1895564

**Published:** 2026-09-17

**Authors:** Rekha Rani, Shivam Kumar, Akhil Singh, Rachapudi Venkata Sreeharsha

**Affiliations:** Biofuels and Climate Change Mitigation Laboratory, Department of Life Sciences, Chhatrapati Shahu Ji Maharaj (CSJM) University, Kanpur, India

**Keywords:** 6-benzylaminopurine, *Cajanus cajan* L., chlorophyll a fluorescence, drought tolerance, gibberellic acid, metabolome, proton motive force, transcriptome

## Abstract

**Introduction:**

Drought stress adversely affects photosynthesis, redox homeostasis and metabolic signalling in plants. Exogenous application of hormonal and nonhormonal elicitors, have emerged as promising strategies to improve plant resilience to changing climatic conditions.

**Methods:**

In the current study, the effect of foliar application of gibberellic acid (GA_3_) and 6–benzylaminopurine (6–BAP) individually and in combination was assessed at different doses (0.2 and 0.4 mM) in pigeonpea (*Cajanus cajan*) under drought stress through integrated physiological, metabolomic and transcriptomic approaches.

**Results:**

Growth, photosynthetic performance and metabolic homeostasis were severely affected by drought stress. The combined application of 0.4 mM GA3+6-BAP resulted in 20.6%, 29.7% and 33.2% increase in the maximum quantum efficiency of PSII (Fv/Fm), ΔpH, and ΔΨ, respectively, compared to untreated drought-stressed plants, which shows that the stability of PSII and the energy regulation of the thylakoids improved with combined phytohormone application. Untargeted metabolite profiling revealed that drought reduced the abundance of metabolites belonging to carbohydrates, lipids and organoheterocyclic compounds respectively. While GA3+6-BAP led to the restoration of metabolites involved in carbon metabolism and antioxidative metabolism wherein highest accumulation of flavonoids, phenolics and terpenoids was noticed. Hormonal application induced 24 unique metabolic signatures including quercetin 3-(2-glucosylrhamnoside), camellianin A, 3-O-p-coumaroylquinic acid, byakangelicin. Besides, transcriptomic profiling demonstrated treatment-associated changes in the abundance of transcripts involved in photosynthesis, redox regulation and calcium-based signaling, suggesting coordinated stress signaling.

**Conclusion:**

Overall, 0.4 mM GA3+ 6-BAP treatment proved to be the most effective among other treatments for the induction of drought stress tolerance in pigeonpea by dynamic transcriptional and metabolic regulatory mechanisms.

## 1 Introduction

Climate change, associated with abiotic stress, affects plant metabolism to a greater extent. Crop plants manifest wide range of signalling cascades through differential expression of proteins and metabolites in response to unfavourable environmental conditions (Chaudhry and Sidhu, 2022; Wang Y. et al., 2023; Zandalinas and Mittler, 2022). Tissue specific transcriptomic and metabolic repertoire of cells in response to stress conditions, provide essential information to design and develop future ready crops (Yu et al., 2025). Although genome editing represents a promising targeted strategy, its application remains challenging in various crop species. Alternatively, non-genetic, input-based crop improvement approaches including application of growth regulators (Colebrook et al., 2014), nanofertilizers (Rastogi et al., 2019), small peptides (Takahashi et al., 2018), and customized bioformulations (Al-Turki et al., 2023; Batool et al., 2020) show promising results in imparting stress resilience in crop plants. The underlying molecular mechanisms behind the incidental advantage are obscure, nonetheless phytohormones (PHs) are presumed to be involved indirectly in eliciting such favourable responses through activation of the signalling cascade of crucial genes (Takahashi et al., 2018; Peleg and Blumwald, 2011).

Native as well as synthetic phytohormones are known to regulate a multitude of plant processes including seed germination (Tyler et al., 2004), photosynthesis (Cortleven and Schmülling, 2015), growth (Achard et al., 2006) and development (Depuydt and Hardtke, 2011). Required in as low as nanomolar concentrations, PHs act as signalling molecules to precisely regulate complex physiological processes including xylem differentiation (Kondo et al., 2016), and leaf senescence (Guo et al., 2021) to cope with the environmental conditions. They are also believed to provide dosage dependent protection against abiotic and biotic stresses in crop plants through species-specific antagonistic and synergistic cross talk. Endogenous gibberellins (GAs) regulate seed germination, cell elongation, stem growth and reproductive development, while cytokinins (CKs) control cell division, shoot development, nutrient mobilization and maintenance of leaf photosynthetic activity (Castro-Camba et al., 2022; Kieber and Schaller, 2018). It has been seen that drought often affects GA and CK homeostasis, which is usually associated with a reduction in growth-promoting signals as the plant prepares for its survival, and that a decrease in the availability of CK is correlated with an increase in leaf senescence and loss of photosynthetic potential (Iqbal et al., 2022; Wahab et al., 2022). Notwithstanding the endogenous PH signalling, their exogenous application showed intriguingly contradicting and species-specific responses under different environmental conditions (Wani et al., 2016). However, the integrative mechanistic aspects underlying crop responses to exogenous hormonal application at physiological, biochemical and molecular level are yet to be fully understood especially in legumes. Thus, it is hypothesized that the exogenous application of exogenous GA_3_ and 6-benzylaminopurine (BAP), a synthetic cytokinin, might help restore the balance of hormones and maintain growth during stress when used at appropriate concentration.

Pigeonpea (*Cajanus cajan* L.,) is an important legume crop, acting as a source of dietary protein and amino acids (Unnikrishnan et al., 2021; Sreeharsha and Reddy, 2015). It plays a crucial role in nitrogen biogeochemical cycle, by fixing atmospheric N_2_ with the help of rhizobium thereby reducing the need for chemical fertilizers. Owing to its staple nature, breeding and conservation of its genetic resources has garnered interest in the recent past. Despite its inherent resilience, pigeonpea reproductive development and productivity are substantially constrained due to environmental constraints including drought stress (Choudhary et al., 2011; Unnikrishnan et al., 2024). Understanding the effects of phytohormones at molecular level under stress conditions and the genetic basis underlying the phenological responses is critical to identify candidate genes and pathways for developing strategies to improve their tolerance. Additionally, there is a pressing need to create metabolite libraries for major pulses, which was currently at a nascent stage, as it was done in the case of cereals and other model species. The current study is aimed at understanding the optimum concentration range of combined natural and synthetic phytohormones, i.e., gibberellic acid (GA_3_) and benzylaminopurine (6-BAP) for exogenous foliar applications to induce growth promotion and stress resilience. We hypothesize that combined application of GA_3_ and 6-BAP would bring about coordinated physiological and molecular responses associated with enhanced photosynthetic efficiency and drought tolerance in pigeonpea. We employed an integrative physiological and multi-omics framework to determine the impact of different concentrations of GA_3_ and 6-BAP on drought resilience in pigeonpea.

## 2 Materials and methods

### 2.1 Experimental layout

A commercial pigeonpea variety (IPA 15-2; Sharada) was grown in shady net facility of Chhatrapati Shahu Ji Maharaj University (Latitude: 26°30′N; Longitude: 80°16′E; altitude:318 m above mean sea level), Kanpur, India. Seeds were surface sterilized with 0.1% sodium hypochlorite for 10 min and sown in 12-inch terracotta pots filled with a homogenized soil: farm yard manure mixture (2:1), and maintained under uniform irrigation. All plants were maintained for 180 days after emergence (DAE) for complete life cycle till seed harvest. For hormonal treatments, two-month-old healthy plants at vegetative stage were selected. A total of fourteen treatments including control were formulated in triplicates, to study the effects of hormonal regulation on drought stress. Different hormonal combinations include individual and combined formulations of gibberellic acid (GA_3_) and benzylaminopurine (6-BAP) applied at 0.2 mM and 0.4 mM concentrations, while the control plants were sprayed with distilled water (mock) without any hormone (Table 1). Drought stress was imposed by completely withholding irrigation and drought severity was determined by measuring leaf relative water content (LRWC).

**TABLE 1:** Experimental Layout with different hormone and drought stress combinations. Mock: hormone-free distilled water spray; WW: well-watered; D: drought.

Treatment	Phytohormone	Concentration	Stress
**T-1**	None	Mock	WW
**T-2**			D
**T-3**	GA_3_	0.2 mM	WW
**T-4**			D
**T-5**		0.4 mM	WW
**T-6**			D
**T-7**	6-BAP	0.2 mM	WW
**T-8**			D
**T-9**		0.4 mM	WW
**T-10**			D
**T-11**	GA_3_ + 6-BAP	0.2 mM	WW
**T-12**			D
**T-13**		0.4 mM	WW
**T-14**			D

The first exogenous application of phytohormone was carried out at 60 DAE, followed by a second spray at 67 DAE. Additionally, drought–hormone interactions were studied by imposing drought stress from 68 DAE on the designated hormone-treated plants by completely withholding water for a period of 8 days, during which drought symptoms were confirmed by visible wilting and reduced LRWC compared with well-watered plants, resulting in GA_3__D, 6-BAP_D, and GA_3_+6-BAP_D at 0.2 mM and 0.4 mM concentrations (Table 1). Corresponding control plants were irrigated regularly. At the end of the drought treatment, physiological, biochemical, and growth-related measurements were recorded for all treatments. Plant samples were collected and immediately snap-frozen in liquid N_2_, and stored at −80 °C for subsequent metabolomic, transcriptomic, and biochemical analyses.

### 2.2 Growth, morphology and biochemical analysis

Morphological parameters including plant height, number of primary branches, and leaf area, were recorded. Plant height (cm) was measured using a measuring tape from the upper surface of the pot to the apical tip of the plant. The number of primary branches was counted manually. Leaf area (cm^2^) was determined using the graphical method by tracing the leaf outline onto graph paper and counting the enclosed squares. For biomass measurements, plants from each treatment were carefully uprooted at the end of the growing season, washed to remove adhering soil, and separated into leaf, stem, and root fractions. Fresh weight of each fraction was recorded immediately using an analytical balance. Samples were then oven-dried at 60 °C ± 2 °C to constant weight and dry biomass (g plant^-1^) was calculated. The root–shoot ratio was computed to assess biomass allocation patterns under hormonal and stress treatments. LRWC was determined by taking fresh weight (FW) of fully expanded leaves immediately after excision. Leaves were then floated on double-distilled water (DDW) for 24 h at room temperature to obtain full turgidity, after which turgid weight (TW) was measured. Samples were subsequently oven-dried at 60 °C to constant weight to determine dry weight (DW). Relative water content was calculated using the formula; RWC (%) = (FW-DW)/(TW-DW) *100.

Photosynthetic pigments were extracted using dimethyl sulfoxide (DMSO) (Hiscox and Israelstam, 1979). Fresh leaf discs were incubated in DMSO until complete pigment diffusion. The absorbance of the extract was recorded at 663.2, 646.8, and 470 nm using a UV–Visible spectrophotometer (Genesys 180, Thermo Scientific, USA). Chlorophyll a (Chl a), chlorophyll b (Chl b), and total carotenoids were calculated using the equations derived from the extinction coefficients and expressed on a fresh weight basis. Chl a (µg mL^-1^) = 12.25 × A_663.2_–2.79 × A_646.8_; Chl b (µg mL^-1^) = 21.50 × A_646.8_–5.10 × A_663.2_; Total carotenoids (µg mL^-1^) = (1,000 × A_470_–1.82 × Chl a −85.02 × Chl b).

Anthrone method was used to estimate the total soluble carbohydrates using glucose standard curve (Hedge and Hofreiter, 1962). Leaf sample (100 mg) was subjected to hydrolysis, reaction with anthrone reagent and measuring the absorbance at 630 nm. The Bradford assay was used to determine the total soluble protein. Leaf sample (200 mg) was extracted in phosphate buffer and the absorbance of the solution at 595 nm was recorded as described elsewhere. The concentration of the protein was determined using a BSA standard curve and reported as mg g^-1^ FW (Bradford, 1976).

To determine C:N ratio, individual elemental composition including carbon (C), hydrogen (H), nitrogen (N), and sulfur (S) were determined using combustion-based CHNS analysis. Oven-dried leaf samples (65 °C to constant weight) were finely ground, and approximately 5 mg of each sample was packed in tin capsules and analyzed using an elemental analyzer (Unicube, Elementar, GmbH, Germany). Samples underwent dynamic flash combustion (∼1,150 °C) in an oxygen-rich atmosphere, and the resulting gases were separated chromatographically and detected using a thermal conductivity detector (TCD). The instrument was calibrated at the beginning of each analytical batch using certified sulfanilamide as the calibration standard. Calibration was verified periodically throughout the analytical batch using the certified reference material, and blank tin capsules were analysed as quality control. According to the manufacturer’s specifications, the analytical precision of the instrument was better than ±0.1% (absolute) for homogeneous samples. Results were expressed on a dry-weight basis (%C, %H, %N, %S), and C:N ratios were calculated accordingly (Sreeharsha et al., 2025).

For measuring hexose: sucrose (Hex: Suc) ratio, extracted soluble sugars were analysed using Ultra High-Performance Liquid Chromatography (UHPLC) separation. Briefly, fresh leaves (200 mg) were extracted using 5 mL of 80% (v/v) ethanol at 60 °C–70 °C for 30 min, followed by centrifugation (10,000 rpm, 10 min) using refrigerated centrifuge (DLab Scientific, China). The supernatant was concentrated to ∼2 mL, and then filtered through a 0.22 µm membrane filter. Sugar quantification was performed using a UHPLC system (Thermo Fisher Scientific, USA) equipped with a refractive index (RI) detector and a Thermo Scientific™ Hypersil™ NH_2_ amino column (250 × 4.6 mm, 5 µm). Separation was carried out under isocratic conditions using acetonitrile, water (75:25, v/v) as the mobile phase at a flow rate of 1.0 mL min^-1^, with an injection volume of 10 µL and column temperature maintained at 30 °C. Glucose, fructose, and sucrose were identified against authentic standards (Sigma Aldrich, USA) and quantified using external calibration curves (*R* ^2^ ≥ 0.99) and Hex: Suc ratio was subsequently calculated (Sreeharsha et al., 2019).

### 2.3 Transient kinetics of photosystem II and proton motive force (PMF) of thylakoid membranes

Chlorophyll *a* fluorescence parameters were measured on the adaxial surface of fully expanded leaves which were pre dark adapted for 20 min using a portable MINI-PAM fluorometer (Heinz Walz, GmbH, Effeltrich, Germany). Quantum yield measurements were obtained by generating light response curves through stepwise increases in actinic photosynthetically active radiation (PAR), ranging from 0 to 1,500 μmol photons m^-2^ s^-1^. Each rapid light curve measurement lasted about 5.5 min, with fluorescence recordings taken sequentially as irradiance increased, followed by the application of a saturating pulse (SP) of 8,000 μmol photons m^-2^ s^-1^ at equal interval of time (30s) to determine maximum fluorescence in the light-adapted state (Fm′).

Chlorophyll *a* fluorescence was also measured using a slow induction kinetics program of Dual-PAM-100 system (Heinz Walz GmbH, Effeltrich, Germany) to understand photoinhibition patterns of control and treated plants upon exposure to continuous high light. Dark-adapted leaves were exposed to continuous AL (569 μmol photons m^-2^ s^-1^) for 10 min, with SP (8,000 μmol photons m^-2^ s^-1^) applied at 50 s intervals to monitor time-dependent changes in photochemical and non-photochemical quenching. Relaxation kinetics were recorded during a 10-min dark recovery phase following cessation of AL, using SP of 8,000 μmol photons m^-2^ s^-1^ to quantify NPQ decay dynamics (Schreiber et al., 1986).

Further, to study the proton motive force of thylakoid membrane under stress and hormonal treatments, the dual-beam 550–515 nm difference signal was recorded using the P515/535 module integrated with the Dual-PAM-100 system (Heinz Walz GmbH, Germany). Plants were dark-adapted for 30 min prior to measurements; leaf was exposed to measuring light for 30s and actinic light (569 μmol photons m^-2^ s^-1^) was applied after 30 s of baseline recording and switched off after 300 s for 180 s to determine proton motive force (pmf) components through kinetic analysis of ECS relaxation. Electrochromic shift (ECS) signals reflecting changes in membrane potential (Δψ) and proton gradient (ΔpH), as well as zeaxanthin-dependent components, were analyzed from dark–light–dark induction transients (Dhokne et al., 2022).

### 2.4 Metabolite profiling through liquid chromatography coupled with high resolution mass spectrometry (LC-HRMS)

Fresh leaves (100–150 mg) from each biological replicate were immediately snap-frozen in liquid N_2_ and preserved at −80 °C until further use. For extraction, frozen leaves were ground into a fine powder in liquid N_2_ and extracted using ice cold methanol (80%) with continuous stirring. Samples were incubated on ice for approximately 30 min followed by ultrasonication for 10 min pulsed mode (30s on/30 s off). The extracts were centrifuged at 12,000 rpm for 10 min at 4 °C using a refrigerated centrifuge (DLAB Scientific, China), and the resulting supernatants were filtered through a 0.22 µm PTFE membrane. The clarified extracts from three biological replicates were combined in equal amounts and used for LC-MS profiling. The LC-HRMS was performed using an Agilent 6540 Q-TOF MS with a Dual Agilent Jet Stream electrospray ionization source (positive mode, m/z 100–1700). Chromatographic separation was achieved on an Agilent Eclipse XDB-C18 (3 × 150 mm, 3.5 µm) maintained at 40 °C with mobile phases A (0.1% formic acid in water) and B (0.1% formic acid in acetonitrile) at 0.3 mL min^-1^ using the specified gradient (0–2 min 95/5 A/B; 2–25 min to 5/95; 25–28 min 5/95; 28.1–30 min 95/5). Full-scan data were acquired from m/z 100–1700 under the provided source conditions (nebulizer gas temperature 300 °C, sheath gas temperature 350 °C, drying gas 8 L min^-1^, nebulizer 35 psig (pressure), sheath gas 11 L min^-1^, capillary 3500 V, nozzle 1000 V). Raw data were processed in Agilent MassHunter for molecular feature extraction, deconvolution, retention time alignment, and compound grouping across samples (Agilent MassHunter Profinder documentation). The features were then filtered, normalized (m/z–retention time pairs with integrated peak areas) and log-transformed. The metabolite abundance data were auto-scaled (unit variance scaling) before analysis. Principal component analysis (PCA) and hierarchical clustering analysis (HCA) were conducted using R (version 4.5.2) and univariate analysis was used to determine the metabolites that were differentially abundant among treatments. Putative metabolite annotation was carried out by matching accurate masses against public databases, including METLIN, HMDB and KEGG, and the identified metabolites were reported as putatively annotated. (Theodoridis et al., 2008).

### 2.5 RNA-seq mediated differential gene expression analysis

Total RNA was extracted from fresh leaves of three biological replicates of all the treatments using Spectrum Total Plant RNA isolation kit (Sigma Aldrich, USA). High quality RNA with RNA Integrity Number (RIN) greater than eight was used for library preparation after controlled fragmentation of mRNA. First-strand cDNA synthesis was performed using random hexamer primers, and second-strand synthesis generated double-stranded cDNA using manufacturer’s instructions. Equimolar concentrations of RNA from the three biological replicates within each treatment were pooled prior to library preparation. After end repair, A-tailing, and adapter ligation, libraries were PCR-amplified, size-selected, and quality-validated prior to sequencing. Paired-end RNA sequencing was performed on an Illumina NovaSeq platform (Illumina, San Diego, CA, USA) to generate high-depth transcriptomic data. Upon sequencing, raw reads were subjected to quality assessment using FastQC and adapter sequences and low-quality bases were removed using fastp (V0.23.4). Sequence quality was evaluated using Phred quality scores (Qphred = −10 log_10_^e^), and high-quality clean reads meeting Q20 and Q30 thresholds were retained for downstream analyses (Chen et al., 2018). *De novo* transcriptome assembly was performed using Trinity (v2.15.2), which reconstructs transcripts based on a de Bruijn graph algorithm. The initial Trinity assembly generated 275,048 contigs with an N50 of 2,350 bp and a GC content of 38.51%. To eliminate redundant transcripts and improve downstream functional analyses, clustering was conducted using CD-HIT, resulting in a refined non-redundant transcriptome consisting of 94,296 contigs with an N50 of 2,328 bp and GC content of 38.33%. To assess assembly completeness and transcript representation, clean paired-end reads were aligned back to the non-redundant transcriptome using HISAT2, and alignment files were processed using SAM tools (Kim et al., 2019; Li and Godzik, 2006). Alignment rates ranged from 93.55% to 99.90%, indicating high-quality assembly and comprehensive transcript recovery. Transcript abundance was quantified using SALMON, which applies a quasi-mapping–based probabilistic model to estimate transcript-level expression while correcting for sequence-specific and GC-content biases. Differential gene expression analysis was performed using the EdgeR (v4.2.2) package in R by applying thresholds of |log_2_FC| ≥ 2 and p ≤ 0.05, with multiple-testing correction conducted using the Benjamini–Hochberg false discovery rate (FDR) to control type I errors (Patro et al., 2017). Raw count data were normalized using the trimmed mean of M-values (TMM) method to account for library size variation. Statistical testing was conducted using a negative binomial generalized linear model framework. To control for multiple hypothesis testing, p-values were adjusted using the Benjamini–Hochberg false discovery rate (FDR) correction. Genes exhibiting an adjusted FDR ≤0.05 and log_2_ fold change ≥2 were considered as significantly differentially expressed. Pairwise comparisons were conducted among treatment groups to identify drought-responsive and hormone-modulated transcriptional changes. Functional annotation of assembled transcripts was performed using BLASTx against the UniProt protein database to identify homologous sequences and assign putative functional descriptions. Orthology-based annotation was further performed using EggNOG to assign Gene Ontology (GO), COG classifications, and KEGG pathway association (Huerta-Cepas et al., 2019). Gene Ontology enrichment and KEGG pathway analyses were conducted using the gProfiler (V0.2.3) and R package was used to identify significantly overrepresented biological processes and metabolic pathways among differentially expressed genes.

### 2.6 qRT PCR analysis

Quantitative real-time PCR (qRT–PCR) was performed for selected crucial genes to validate gene expression patterns obtained from RNA-Seq analysis. First-strand cDNA was synthesized from 1 µg of total RNA using a reverse transcription kit following the manufacturer’s protocol (Thermo Scientific, USA). Gene-specific primers were used to amplify target genes, and primer specificity was verified by melt-curve analysis. qRT–PCR reactions were performed using SYBR Green (Thermo Scientific, United States) chemistry in a real-time PCR detection system (Applied Biosystems, USA). Each sample included three biological replicates with three technical triplicates. Relative transcript abundance was normalized against the stable internal reference gene actin, and expression levels were calculated using the 2^−ΔΔCT^ method (Livak and Schmittgen, 2001), where ΔC_t_ = C_t_ (target gene) – C_t_ (reference gene) and ΔΔC_t_ = ΔC_t_ (treatment) – ΔC_t_ (control). Results were expressed as fold change relative to the control treatment.

### 2.7 Network analysis

Pearson correlation analysis was used to construct gene–metabolite association networks. Absolute Pearson correlation coefficient (|r| ≥ 0.80) and P < 0.05 were considered significant and included in network construction. The association network was visualized using the Cytoscape v3.10.4. The topology of the network was analyzed using the NetworkAnalyzer tool in Cytoscape and the hub genes (genes with the highest number of direct connections) were identified based on degree centrality.

### 2.8 Statistical analysis

All experiments were conducted with three independent biological replicates, each consisting of technical triplicates unless otherwise stated. Treatment effects were evaluated using one-way (ANOVA), depending on the experimental design, followed by Tukey’s HSD test. Statistical significance was accepted at *P* < 0.05. In addition, two-way ANOVA was performed to assess the main effects of hormone treatment (H), drought (D), and their interaction (H × D). The F-values and significance levels are shown in the figure panels, wherever applicable. All analyses were performed and plotted using GraphPad Prism (GraphPad Software, USA).

## 3 Results

### 3.1 Growth and biochemical responses under drought and hormone treatments

Progressive drought stress and exogenous GA_3_ and 6-BAP application significantly influenced growth, biomass accumulation, and biochemical traits in pigeonpea (Figure 1). Furthermore, phytohormones alleviated the morphological and biochemical symptoms of drought, though the magnitude of the response differed among hormone concentrations. Among the individual treatments, 0.4 mM GA_3_ resulted in highest plant height (*F_*41.54; *P* < 0.001) and leaf area (F_61.94; *P* < 0.001) under drought conditions compared to well-watered control plants, outperforming the 6-BAP and its combined treatments (Table 2). In contrast, the combined hormonal application produced significant increase in branch number and biomass accumulation, irrespective of irrigation regime (Figure 1; Table 2). Furthermore, LRWC, an indicator of plant water status, declined markedly under drought stress reaching a minimum of 33.67 ± 1.12, after 8 days of stress. In contrast, GA_3_ and 6-BAP treatments maintained relatively higher LRWC under both well-watered and drought conditions. Specifically, 0.4 mM GA_3_ treated leaves maintained LRWC of 57.74 ± 0.72 while, the corresponding combined hormone treatments with same molar concentration maintained LRWC of 58.09% ± 2.30% at the end of the drought period, indicating the role of 0.4 mM GA_3_ and 6-BAP application in sustaining leaf water status during the drought regimes (Table 2). In addition, drought stress significantly declined vegetative and reproductive biomass by 49% and 68% respectively compared with irrigated plants. Whereas, individual GA_3_ and 6-BAP application increased the biomass production under drought conditions, wherein the increase was more prominent when they were given in 0.4 mM combination (Table 3). The biochemical analysis further demonstrated significant treatment-dependent differences in primary metabolites and photosynthetic pigments (Figure 2). The combined application of 0.4 mM GA_3_+6-BAP, pigeonpea leaves exhibited the highest carbohydrate accumulation (13.14 ± 0.77 mg g^-1^ FW) and proteins (1.11 ± 0.01 mg g^-1^ FW) among the treatments. Likewise, the chlorophyll and carotenoid contents were higher by 35% and 29% respectively in 0.4 mM GA_3_ treated plants as compared to mock (Figure 2). Furthermore, the C/N ratio calculated as a fraction of inorganic elemental composition also increased significantly in the drought-stressed plants, while it was comparatively low in the hormone treated plants, especially in combination (Figure 2). Hex/Suc ratio was found to be increased under drought conditions irrespective of hormonal treatment and found to be further enhanced by GA_3_ treatment (Figure 2). The results indicate that the 0.4 mM hormone treatments, particularly the combined application of GA_3_ and 6-BAP, consistently produced improved growth, biomass accumulation, leaf water status, and biochemical characteristics under drought stress.

**FIGURE 1:**
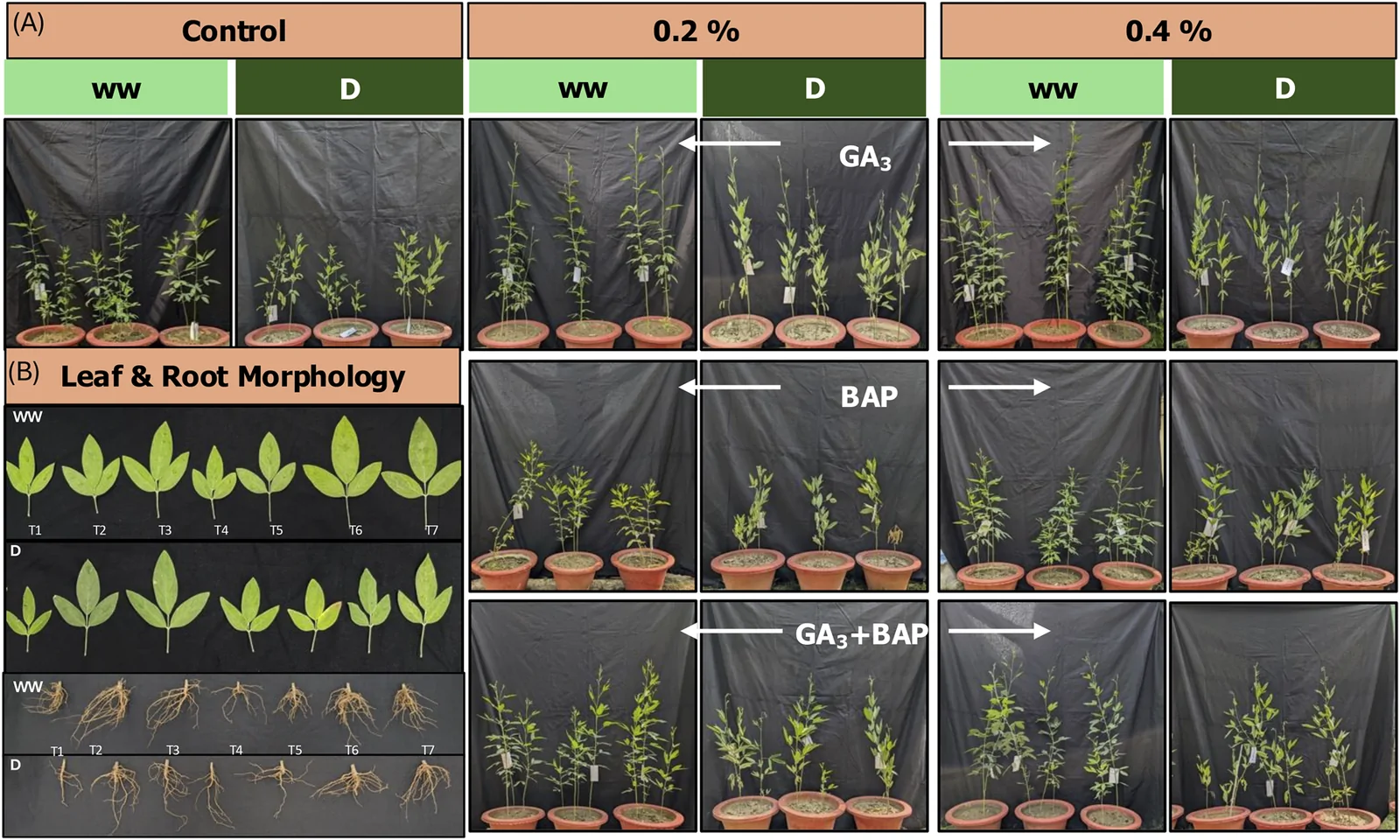
Experimental design and morphological responses of pigeonpea. Representative images showing the experimental layout and morphological responses of mock control (hormone-free distilled water spray), drought-stressed, hormone-treated (GA_3_, BAP, and GA_3_+BAP at 0.2 and 0.4 mM concentrations), and interactive hormone and drought-stressed plants. **(A)** Whole-plant morphology under the different treatment conditions **(B)** Leaf and root morphology. WW, well-watered; D, drought; GA_3_, gibberellic acid; BAP, 6-benzylaminopurine; GA_3_+BAP, combined application of gibberellic acid and 6-benzylaminopurine.

**TABLE 2:** Effect of hormone treatments on growth and water status parameters.

Treatment	Water status	PH (cm)	LA (cm^2^)	Branches	LRWC (%)
Control	WW	89.67 ± 8.74^c^	5.00 ± 0.50^d^	3.33 ± 0.58 ^cd^	71.98 ± 0.74^c^
	D	67.00 ± 2.65^f^	4.13 ± 0.23^e^	2.33 ± 0.58^d^	33.67 ± 1.12^e^
0.2 GA	WW	132.33 ± 7.51^a^	6.17 ± 0.29^c^	7.33 ± 1.15 ^ab^	76.89 ± 0.98^d^
	D	121.67 ± 7.64 ^ab^	8.33 ± 0.29 ^b^	3.67 ± 1.15 ^cd^	57.96 ± 2.06^a^
0.4 GA	WW	139.33 ± 5.13^a^	7.83 ± 0.29 ^b^	6.67 ± 0.58 ^b^	72.34 ± 0.22^c^
	D	119.67 ± 4.51 ^ab^	8.67 ± 0.58 ^b^	2.67 ± 0.58^d^	57.74 ± 0.72^a^
0.2 BAP	WW	72.00 ± 7.21^e^	5.17 ± 0.29^d^	5.00 ± 1.00 ^bc^	79.14 ± 0.31^c^
	D	66.33 ± 1.53^f^	5.33 ± 0.29^d^	3.67 ± 0.58 ^cd^	32.42 ± 3.61^e^
0.4 BAP	WW	82.67 ± 2.52^d^	7.17 ± 0.29 ^b^	4.67 ± 0.58^c^	74.98 ± 0.72 ^b^
	D	75.67 ± 5.13^e^	5.50 ± 0.50^d^	5.00 ± 0.00^c^	38.04 ± 3.20^c^
0.2 GA + BAP	WW	96.00 ± 4.00^c^	10.83 ± 0.29^a^	4.33 ± 0.58^c^	78.91 ± 0.66^c^
	D	94.00 ± 5.29^c^	6.17 ± 0.29^c^	2.67 ± 0.58^d^	39.39 ± 1.10^c^
0.4 GA + BAP	WW	115.00 ± 5.00 ^b^	11.67 ± 0.58^a^	9.00 ± 1.00^a^	83.77 ± 0.82^a^
	D	113.33 ± 5.77 ^b^	8.17 ± 0.29 ^b^	6.67 ± 1.53 ^b^	58.09 ± 2.30^a^

Plant height (PH) (cm), leaf area (LA) (cm^2^), number of branches, and leaf relative water content (LRWC, %) were measured under different treatments. Data are presented as mean ± SD (n = 3). Data were analyzed using one-way ANOVA followed by Tukey’s HSD test. Means sharing one or more lowercase letters are not significantly different, whereas means with no lowercase letters in common differ significantly (P < 0.05).

**TABLE 3:** Effect of hormone treatments on biomass and yield attributes.

Treatment	Water status	Root FW (g)	Root DW (g)	Shoot FW (g)	Shoot DW (g)	Fruit FW (g)	Fruit DW (g)
Control	WW	5.15 ± 0.04^c^	2.32 ± 0.03^c^	31.13 ± 0.09^c^	15.98 ± 0.05^c^	11.14 ± 0.12^c^	4.59 ± 0.08^c^
	D	2.32 ± 0.02^f^	1.12 ± 0.02^e^	16.68 ± 0.12^f^	8.22 ± 0.18^e^	2.93 ± 0.05^f^	1.47 ± 0.03^e^
0.2 GA	WW	7.73 ± 0.03^a^	3.16 ± 0.02 ^b^	35.26 ± 0.04 ^b^	17.64 ± 0.03 ^b^	11.26 ± 0.05^c^	5.73 ± 0.15 ^b^
	D	3.97 ± 0.03^e^	1.94 ± 0.05^d^	22.55 ± 0.05^d^	11.27 ± 0.04^d^	4.43 ± 0.18^d^	2.17 ± 0.02^d^
0.4 GA	WW	7.96 ± 0.03^a^	3.02 ± 0.03 ^b^	38.64 ± 0.13^a^	16.85 ± 0.03^c^	13.09 ± 0.02 ^b^	6.54 ± 0.05^a^
	D	2.95 ± 0.02^f^	1.27 ± 0.01^e^	24.70 ± 0.05^d^	12.33 ± 0.03^d^	4.78 ± 0.09^d^	2.36 ± 0.03^d^
0.2 BAP	WW	4.82 ± 0.03^d^	1.85 ± 0.03^d^	17.62 ± 0.14^e^	8.82 ± 0.04^e^	10.89 ± 0.09^c^	5.47 ± 0.03 ^b^
	D	3.34 ± 0.02^e^	1.61 ± 0.01^d^	15.63 ± 0.04^f^	7.78 ± 0.07^e^	3.74 ± 0.04^e^	1.87 ± 0.01^e^
0.4 BAP	WW	4.21 ± 0.02^e^	2.96 ± 0.03 ^b^	36.50 ± 0.05 ^b^	18.75 ± 0.03^a^	4.91 ± 0.02^e^	2.49 ± 0.02^d^
	D	3.12 ± 0.02^e^	1.38 ± 0.03^e^	20.72 ± 0.03^e^	10.29 ± 0.07^d^	3.39 ± 0.05^e^	1.76 ± 0.04^e^
0.2 GA + BAP	WW	6.16 ± 0.02 ^b^	5.05 ± 0.03^a^	37.07 ± 0.02^a^	18.56 ± 0.04^a^	11.69 ± 0.30^c^	5.96 ± 0.03 ^b^
	D	2.65 ± 0.01^f^	1.23 ± 0.02^e^	25.14 ± 0.03^d^	13.53 ± 0.05^c^	4.89 ± 0.09^d^	2.51 ± 0.03^d^
0.4 GA + BAP	WW	6.32 ± 0.02 ^b^	2.35 ± 0.02^c^	38.04 ± 0.05^a^	17.65 ± 0.20 ^b^	13.61 ± 0.16^a^	6.49 ± 0.14^a^
	D	4.96 ± 0.02^c^	2.25 ± 0.04^c^	29.91 ± 0.02^c^	16.00 ± 0.09^c^	5.18 ± 0.02^d^	2.62 ± 0.06^d^

Root fresh weight (FW), root dry weight (DW), shoot FW, shoot DW, fruit FW, and fruit DW were measured under different treatments. Data are presented as mean ± SD (n = 3). Data were analysed using one-way ANOVA followed by Tukey’s HSD test. Means sharing one or more lowercase letters are not significantly different, whereas means with no lowercase letters in common differ significantly (P < 0.05).

**FIGURE 2:**
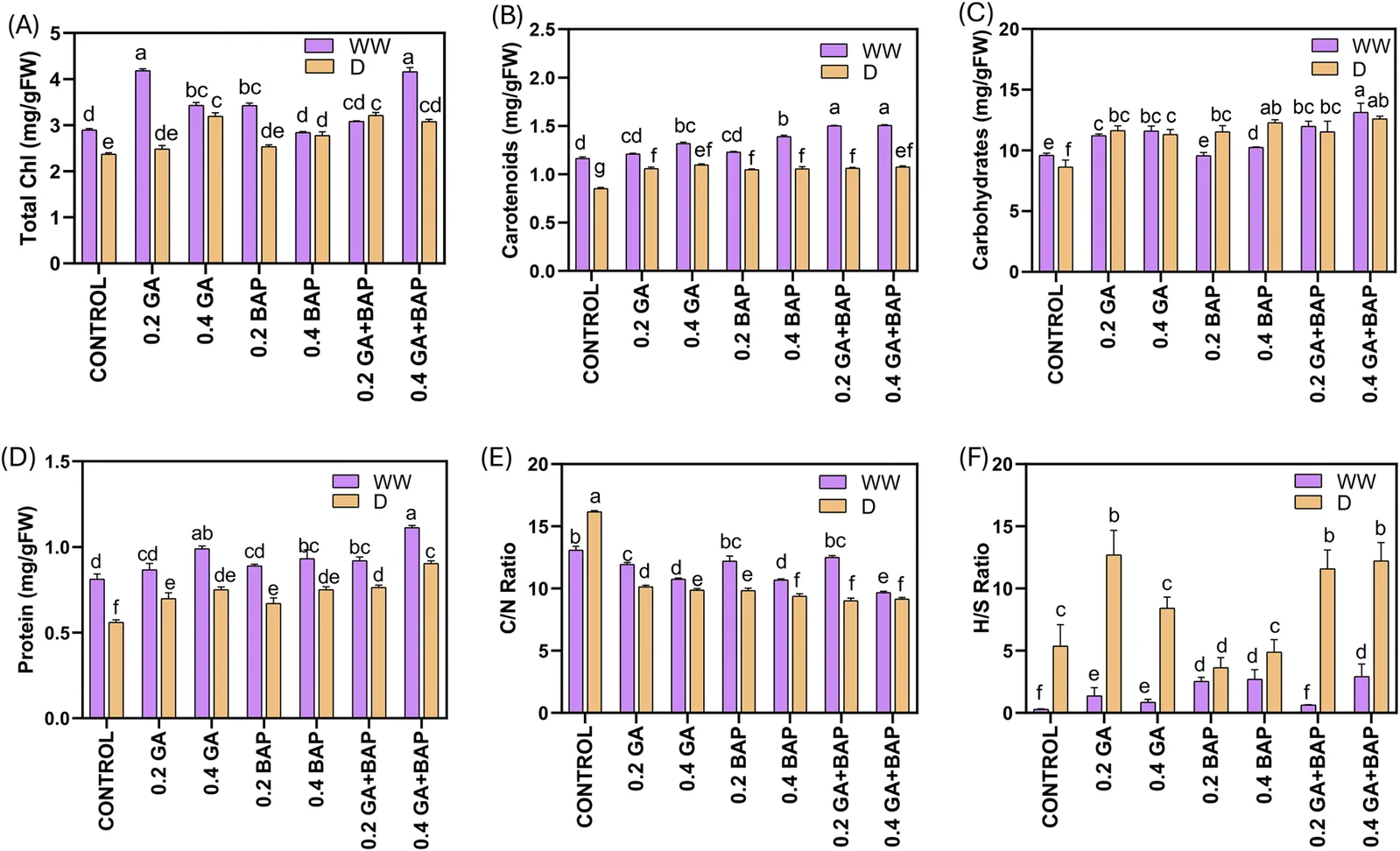
Biochemical responses of pigeonpea leaves treated with GA_3_, 6-BAP, and GA_3_+6-BAP combinations at 0.2 and 0.4 mM under well-watered (WW) and drought **(D)** conditions. **(A)** Total chlorophyll, **(B)** carotenoids, **(C)** carbohydrates, **(D)** protein content, **(E)** C/N ratio, and **(F)** H/S ratio. Data are presented as mean ± SD (n = 3). Statistical difference between means was analyzed using Tukey’s HSD test. Means sharing common lowercase letters are not differed significantly, whereas means with different lowercase letters in common differ significantly at *p* ≤ 0.05.

### 3.2 Dynamics of photosystems under phytohormone and drought treatments

To investigate the physiological basis underlying the observed growth and biochemical responses, chlorophyll fluorescence parameters were measured to evaluate photosynthetic electron transport and photochemical regulation under drought, hormone and their interaction. Chlorophyll fluorescence measurements demonstrated that drought stress reduced both PSII maximum quantum efficiency (Fv/Fm) and electron transport rate (ETR) relative to well-watered plants (Figure 3). Hormone-treated plants maintained higher photochemical performance under drought stress than untreated drought-stressed plants. The combined application of 0.4 mM GA_3_+6-BAP produced the greatest recovery of ETR whereas GA_3_ treated plants maintained comparatively higher Fv/Fm values compared to control plants (Figure 3). Rapid light curve (RLC) analysis revealed distinct treatment-dependent differences in photosynthetic electron transport and energy utilization (Figure 3). The derived parameters obtained through curve fitting of the light response curves using Eilers–Peeters model indicated significant alterations in electron transport capacity and light-use characteristics among the treatments (Figure 3). Drought stress markedly reduced the maximum electron transport rate (ETR*max*) from 93.15 ± 3.01 in well-watered plants to 56.64 ± 1.62. In contrast, foliar application of 0.4 mM GA_3_ resulted in the highest ETR*max* under both well-watered (131.83 ± 4.36) and drought conditions (72.69 ± 2.36) representing increases of 41.51% and 28.34% respectively over their corresponding controls. Similarly, the light saturation parameter (Ek) declined under drought stress but remained comparatively higher in hormone-treated plants, particularly following GA_3_ application. Although the initial slope (α) differed significantly between well-watered and drought treatments, the magnitude of change was relatively small compared with other RLC parameters (Figure 3). Slow induction kinetics of chlorophyll fluorescence also showed significant treatment-dependent variation in photochemical regulation (Figure 3). Drought stress reduced the induction of effective PSII quantum yield Y(II) and ETR, while increasing NPQ relative to mock condition. Hormone-treated plants exhibited higher Y(II) and ETR under drought stress together with comparatively lower NPQ than untreated drought-stressed plants.

**FIGURE 3:**
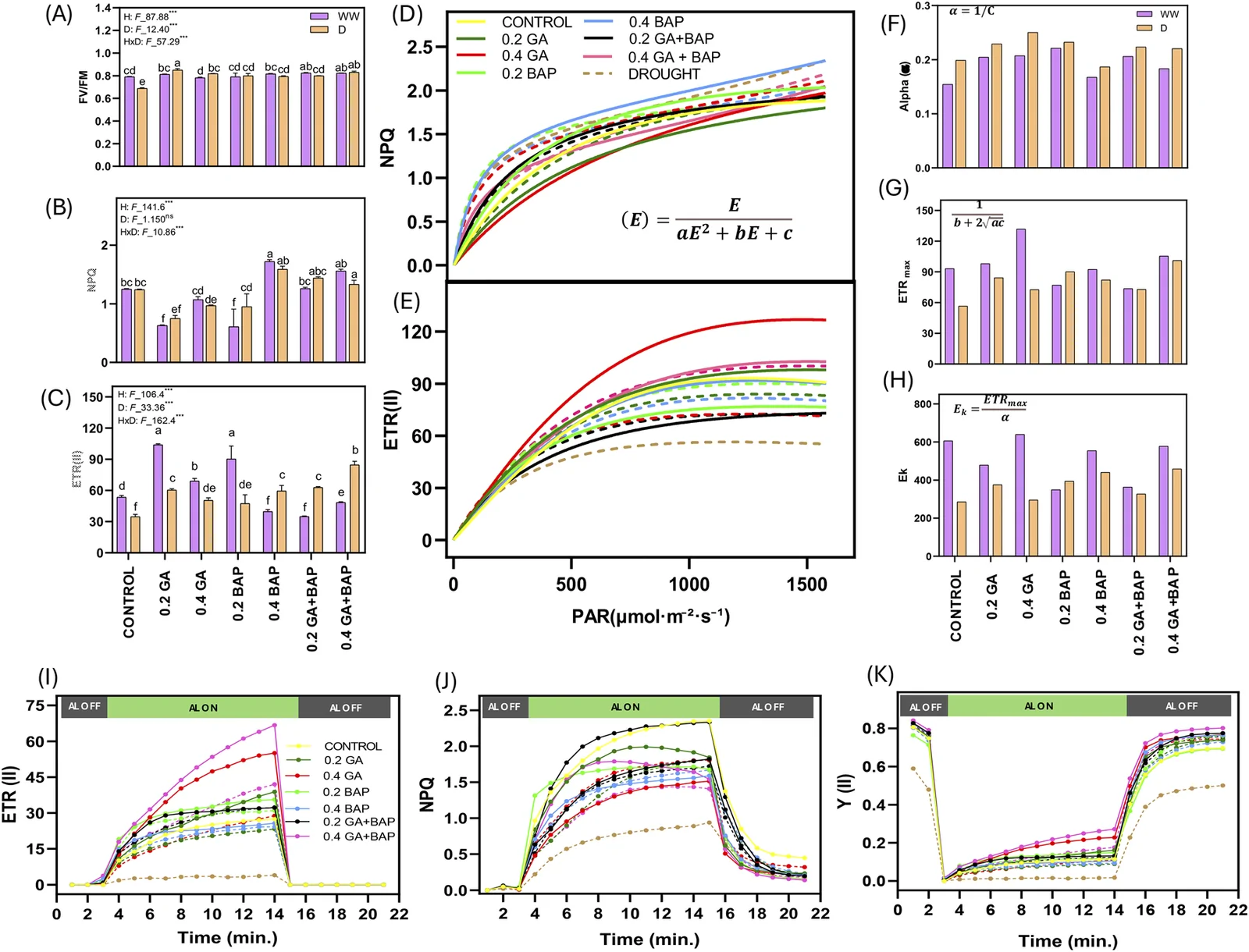
Steady-state chlorophyll *a* fluorescence of pigeonpea measured with mini-PAM under varied conditions **(A)** Maximum quantum efficiency of PSII (Fv/Fm), **(B)** non-photochemical quenching (NPQ), **(C)** electron transport rate (ETR). Also represented chlorophyll *a* fluorescence parameters derived from rapid light curve (RLC) analysis under varied hormonal and drought conditions. **(D,E)** Fitted curves of non-photochemical quenching (NPQ) and electron transport rate (ETR); **(F–H)** ETR derived parameters including maximum electron transport rate (ETR_*max*_), coefficient of low-light induced quenching, derived from initial slope (α), and coefficient of light saturated quenching (Ek). Also represented the photosynthetic parameters derived from slow induction kinetics using Dual-PAM **(I)** PSII electron transport rate [ETR (II)] **(J)** Effective quantum yield of PSII [Y(II)] and **(K)** non-photochemical quenching (NPQ) coloured bars on top of the graph indicates actinic light ON (AL ON), and recovery (AL OFF).

Measurements of proton motive force (pmf) further revealed treatment-dependent changes in thylakoid energy partitioning (Figure 4). Drought stress reduced total pmf, whereas hormone-treated plants maintained higher pmf values under stress. Both the electric potential (Δψ) and proton gradient (ΔpH) components were higher in hormone-treated plants than in untreated drought controls, with the strongest responses observed following GA_3_ and BAP application. These fluorescence responses demonstrate distinct effects of phytohormone treatments on photosynthetic energy utilization and photochemical performance under water-deficit conditions. To further investigate the metabolic changes associated with these photosynthetic responses, metabolomic profiling was performed subsequently with 0.4 mM treated plants.

**FIGURE 4:**
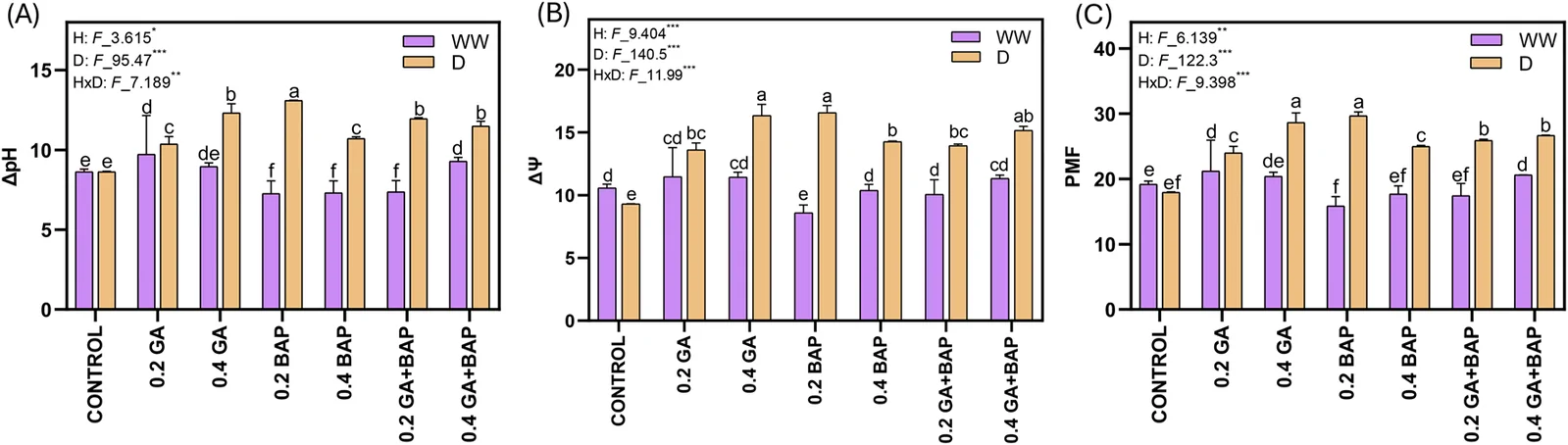
Proton motive force (pmf) parameters **(A)** proton gradient (ΔpH), **(B)** electric potential (ΔΨ), and **(C)** total proton motive force (PMF) in mock control (hormone-free distilled water spray), drought-stressed, hormone-treated (GA_3_, BAP, and GA_3_+BAP at 0.2 and 0.4 mM), and hormone-treated drought-stressed plants under well-watered (WW) and drought (D) conditions. Data are presented as mean ± SD (n = 3). Each biological replicate was measured twice, and the mean value was used for statistical analysis. Data were analyzed using one-way ANOVA followed by Tukey’s HSD test. Means sharing one or more lowercase letters are not significantly different, whereas means with no lowercase letters in common differ significantly (P ≤ 0.05).

### 3.3 Metabolomics revealed differentially regulated metabolites

Metabolomic profiling identified distinct treatment-dependent changes in the metabolite composition of pigeonpea leaves. Untargeted LC-HRMS analysis led to the identification of putative annotations based on database matching. The PCA plot revealed a clear separation among treatments, with PC1 and PC2 explaining 23.8% and 19.6% of total variance, respectively, accounting for cumulative variance of 43.4% (Figure 5). A total of 683 metabolite features were common across all seven comparisons, among which top 270 metabolites were retained for downstream functional interpretation based on their biological relevance and significance threshold. Further, identified metabolite derivatives were annotated with KEGG database to map with different functional categories including primary metabolites (number of metabolites: 45), terpenoids (number of metabolites: 42), lipids and lipid-derivatives (number of metabolites: 48), flavonoids and phenylpropanoids (number of metabolites: 41) (Figure 6). There were 24 metabolite derivatives that appeared in response to hormone application and showed change in abundance when combined with drought treatment. Notably, quercetin 3-(2-glucosylrhamnoside), camellianin A, 3-O-p-coumaroylquinic acid, byakangelicin and PE (14:0/18:2 (9Z,12Z)) indicated hormone-induced metabolic reprogramming in drought adaptation (Supplementary File 1). Metabolites belonging to primary metabolism exhibited marked alterations in response to drought stress. Compared with well-watered plants, drought significantly reduced the relative abundance of metabolites belonging to carbohydrates (58%), lipids (54%), and organoheterocyclic (72%), whereas terpenoid-associated metabolites were comparatively more abundant than control plants (Figure 5). Hormone treatments partially restored these metabolites, with the combined application of GA_3_+6-BAP exhibiting the greatest recovery of lipid- and carbohydrate-associated metabolites (Figure 6). For instance, among lipid-associated metabolites, drought reduced the abundance of compounds including palmitoylethanolamide, palmitic amide, and lysophosphotidylcholine (LysoPC). However, these compounds showed upregulation by 1.51, 0.87 and 2.49 folds with combined hormonal application. Treatment-dependent differences were also observed in carbohydrate- and amino acid-related metabolites (Figure 6). Several phenolic and terpenoid glycoside metabolites including glucocaffeic acid and cyclopassifloside II declined under drought stress but they accumulated in drought treated leaves following GA3+6-BAP application. Recovery of osmolyte- and carbohydrate-associated metabolite signatures under drought was pronounced in plants receiving the combined GA_3_+6-BAP treatment.

**FIGURE 5:**
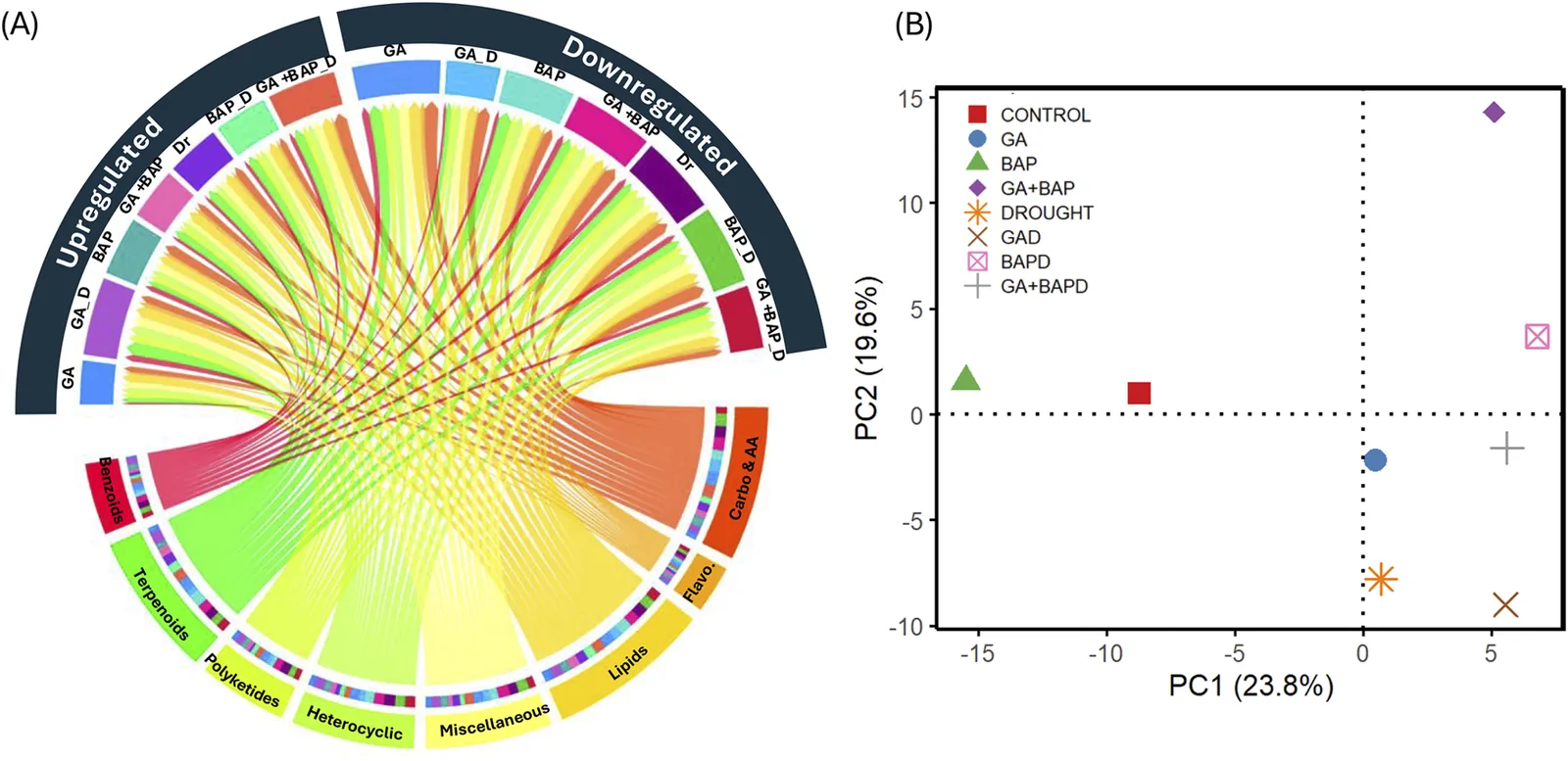
Metabolomic profiling of pigeonpea under hormone treatments and drought stress. **(A)** Chord diagram showing the distribution of upregulated and downregulated differentially accumulated metabolites among major metabolite classes, including benzenoids, terpenoids and polyketides, heterocyclic compounds, miscellaneous metabolites, lipids, flavonoids, and carbohydrates and amino acids (Carbo and AA), based on comparisons of control *versus* drought, control *versus* GA_3_, control *versus* 6-BAP, control *versus* GA_3_+6-BAP, and the corresponding drought-treated comparisons. **(B)** Principal component analysis (PCA) score plot showing the clustering of metabolite profiles from the eight experimental groups comprising mock control, drought, and GA_3_-, 6-BAP-, and GA_3_+6-BAP- treated plants under well-watered (WW) and drought (D) conditions. PC1 and PC2 explained 23.8% and 19.6% of the total variance, respectively (43.4% cumulative variance).

**FIGURE 6:**
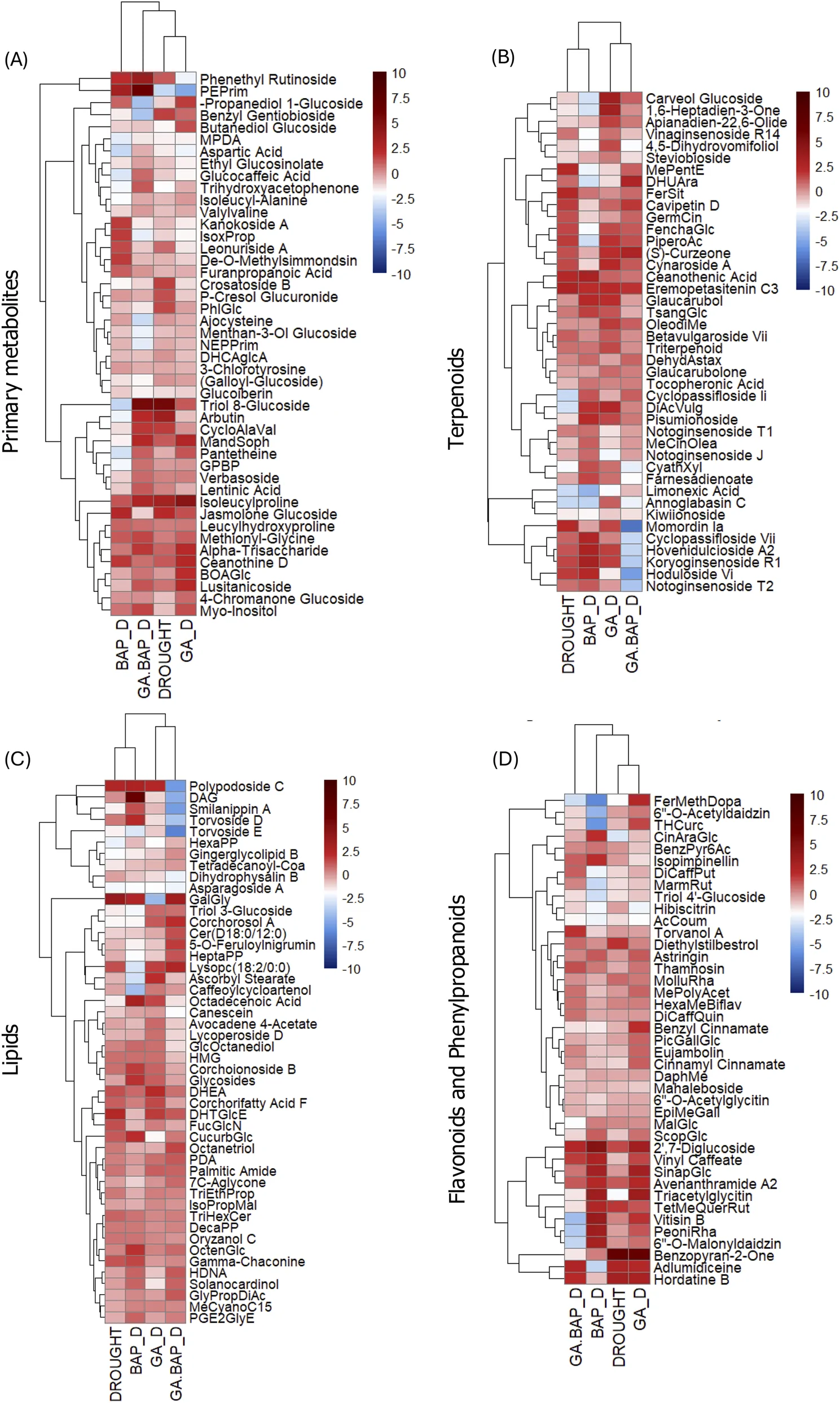
Hierarchical clustering heatmaps of metabolite profiles in pigeonpea under hormone treatments (0.4 mM) and drought stress. Heatmaps showing the accumulation patterns of differentially accumulated metabolites in **(A)** primary metabolites, **(B)** terpenoids, **(C)** lipids, and **(D)** flavonoids and phenylpropanoids across the eight experimental groups comprising mock control (hormone-free distilled water spray), drought, and 0.4 mM GA_3_-, BAP-, and GA_3_+BAP-treated plants under well-watered (WW) and drought **(D)** conditions. Differential metabolites were identified based on log_2_ fold change, false discovery rate (FDR), and statistical significance (P < 0.05). Metabolites were putatively annotated based on accurate mass matching with public databases. Heatmaps were generated using log_2_ fold change values. Red and blue indicate relatively higher and lower metabolite abundance, respectively. Metabolites and samples were hierarchically clustered according to similarities in their profiles.

Secondary metabolite profiling likewise revealed substantial treatment-dependent variation. Drought altered the abundance of several flavonoid, organoheterocyclic, and benzenoid metabolites, whereas hormone treatments generally maintained higher levels of these compounds under drought conditions. Flavonoid and phenolic metabolites accumulated to their highest levels following the combined GA_3_+6-BAP treatment. Similarly, drought altered the abundance of terpenoid metabolites including, limonexic acid and notoginsenoside derivatives, wherein GA_3_ and GA_3_+6-BAP treatments showed specific modulation of their relative abundance. Similar treatment-specific trends were observed for organoheterocyclic metabolites related to indole, adenine and serotonin metabolism, as well as benzoid compounds including gingerenone derivatives and cinnamoyl phenolics, whose relative abundance was differentially modulated by drought and hormone application (Figure 6; Supplementary File 1). Collectively, metabolite profiling demonstrated that phytohormone treatments, particularly the combined application of GA3 and 6-BAP, substantially altered both primary and secondary metabolite profiles under drought stress.

### 3.4 Transcriptome profiling to understand differential gene expression

To determine whether the observed metabolic reprogramming was accompanied by coordinated changes in gene expression, differential transcriptomic analysis was performed. Transcriptome profiling revealed extensive treatment-dependent transcriptional changes in pigeonpea under drought and hormone treatments (Figure 7; Table 4). Differentially expressed genes (DEGs) associated with photosynthesis, carbon metabolism, signal transduction, and redox regulation exhibited distinct expression patterns across the treatments. Among the well-watered plants, 6-BAP treatment produced the highest number of DEGs (26,583), whereas under drought conditions, GA_3_-treated plants exhibited the highest number of DEGs (22,073), indicating extensive transcriptional responses to hormone application (Supplementary File 2). Drought stress reduced the expression of several genes associated with photosynthesis and carbon metabolism, including *RBCS* (−5.13), *STN7* (−3.72) and *ENO* (−4.48). In contrast, genes involved in stress signalling and redox regulation showed increased transcript abundance under drought including the calcium-signalling genes *CNGC2, CRK3, CRK12 and ACA9*, together with the ROS-related genes *APX6, CAT1* and *RBOHC* (Figure 7). Under drought conditions, GA3 application was associated with altered transcript levels of genes involved in carbon assimilation and glycolysis including *RBCS, PFK5, PGM1 *and *ENO*, with *ENO* tshowing higher transcript abundance than in untreated drought-stressed plants. Likewise, the photosynthesis-related gene STN7 also exhibited higher transcript abundance following GA_3_ treatment. Drought-treated plants exhibited strong regulation of transcription factors and signalling genes including *AHL14, WRKY6, ARF4* and *BHLH130, BHLH089* family genes. Under 6-BAP_D treatments, changes in the transcript levels of carbon metabolism-associated genes were less pronounced than under GA_3_ treatments. The combined GA_3_+6-BAP_D treatment exhibited the broadest distribution of fold changes across all functional categories (Figure 7). Compared with the individual hormone treatments, the combined treatment maintained higher expression of genes involved in photosynthesis, carbon metabolism, photochemical regulation, redox regulation, and calcium signalling under drought stress. The transcriptomic analysis demonstrated that phytohormone treatments, particularly the combined application of GA_3_ and 6-BAP, induced extensive transcriptional reprogramming involving photosynthesis, carbon metabolism (*PPD5, PLSB, ENO* and *LPD1*), and redox signalling *(DHAR3, SABP2)* and calcium signalling (*CIPK21, VIP1)* under drought stress.

**FIGURE 7:**
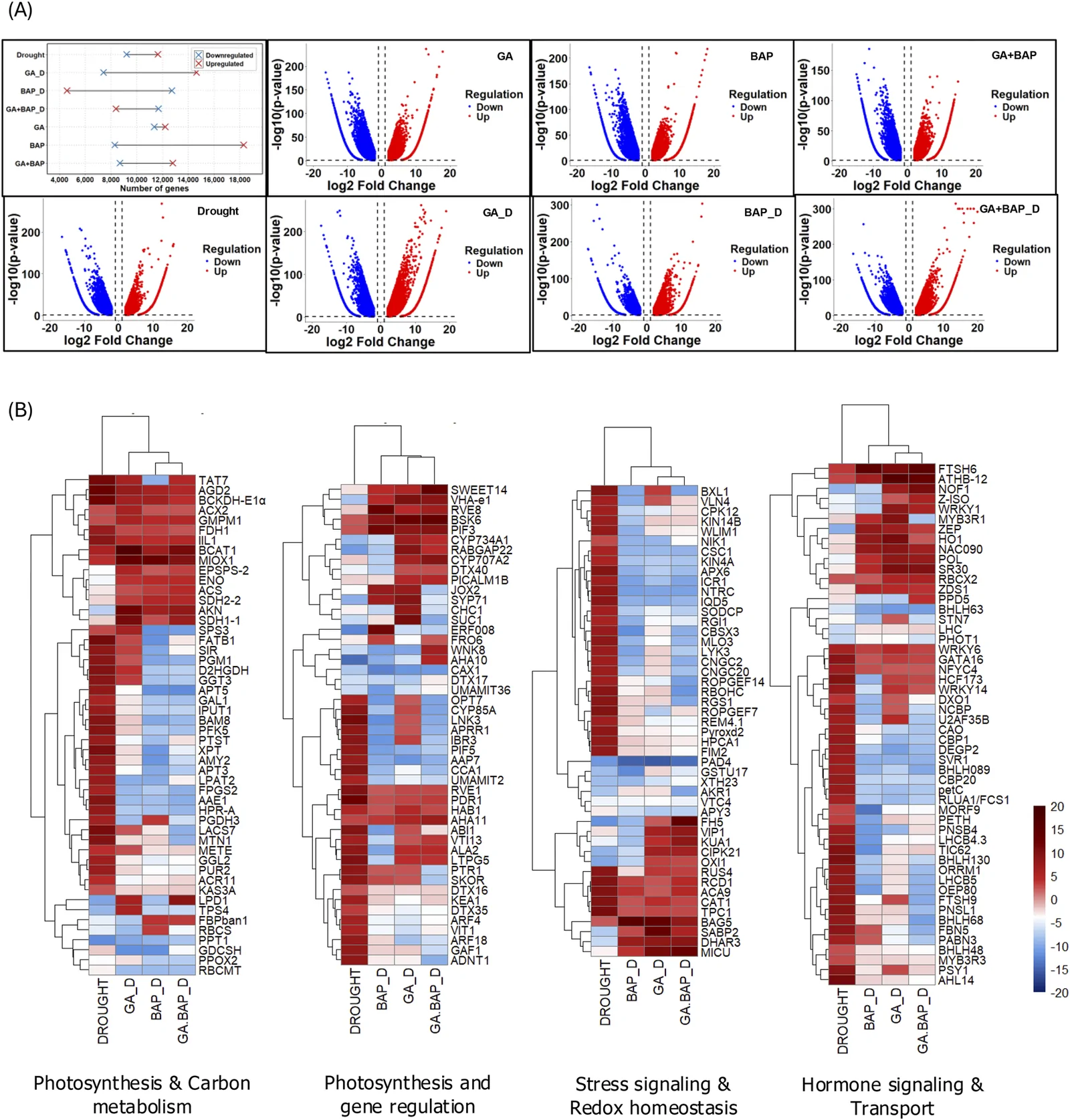
Differential gene expression analysis in pigeonpea under hormone treatments (0.4 mM) and drought stress. Top panel representing **(A)** the number of significantly upregulated and downregulated differentially expressed genes (DEGs) identified in each treatment comparison together with volcano plots showing the distribution of DEGs based on log_2_ fold change and −log_10_ *p* value for the comparisons of control *versus* drought, control *versus* GA_3_, GA_3_ *versus* GA_3__D, control *versus* 6-BAP, 6-BAP *versus* 6-BAP_D, control *versus* GA_3_+6-BAP, and GA_3_+6-BAP *versus* GA_3_+6-BAP_D. Red and blue dots represent significantly upregulated and downregulated genes, respectively, whereas grey dots represent genes that were not differed significantly. Also represented in the lower panel, **(B)** heatmaps of differentially expressed genes under hormone treatments and drought stress. Heatmaps showing the relative expression patterns of genes associated with various functional categories. The fold change was calculated with respect to control *versus* drought, GA_3_ *versus* GA_3__D, 6-BAP *versus* 6-BAP_D, and GA_3_+6-BAP *versus* GA_3_+6-BAP_D. Differentially expressed genes were identified using FDR ≤0.05 and |log_2_ fold change| ≥ 2. The color scale represents relative gene expression levels based on log_2_ fold change, with red indicating upregulated genes and blue indicating downregulated genes. Gene abbreviations are listed in Table 4. Detailed comparisons of mock *versus* hormonal treatments were given in Supplementary File 2.

**TABLE 4:** Treatment-associated differentially expressed genes (DEGs) associated with major metabolic pathways identified by RNA-seq analysis of *Cajanus cajan* leaves from the eight experimental groups comprising mock control (hormone-free distilled water spray), drought, and 0.4 mM GA_3_-, BAP-, and GA_3_+BAP-treated plants under well-watered (WW) and drought (D) conditions. DEGs were identified using FDR ≤0.05 and |log_2_ fold change| ≥ 2.

Abbreviation	Full name	Gene length	Unigenes count	Protein length
Photosynthesis and energy metabolism				
CAO	Chlorophyll a oxygenase	1505	8	338
CBP1	Chloroplast RNA-binding protein 1	3049	10	399
DEGP2	DEG protease 2	1425	8	126
FBN5	Fibrillin 5	1631	9	186
FTSH9	ATP-dependent FtsH metalloprotease 9	3299	3	502
HCF173	High chlorophyll fluorescence 173	2987	17	117
HO1	Heme oxygenase 1	4074	6	203
LHC	Light-harvesting complex protein	971	6	​
LHCB4.3	Light-harvesting chlorophyll a/b-binding protein 4.3	1732	3	175
LHCB5	Light-harvesting chlorophyll a/b-binding protein 5	1713	9	160
MORF9	Multiple organellar RNA-editing factor 9	2051	7	267
OEP80	Outer envelope protein 80	1977	6	560
PETH	Ferredoxin-NADP reductase	1519	9	261
PHOT1	Phototropin 1	2813	10	854
PNSB4	Photosynthetic NDH subcomplex B4	1105	8	117
PNSL1	Photosynthetic NDH subcomplex L1	3774	13	182
PPD5	Photosystem II phosphatase domain protein 5	905	5	​
PSY1	Phytoene synthase 1	2290	3	275
RBCX2	Rubisco accumulation factor RBCX2	6765	7	141
STN7	STN7 thylakoid serine/threonine-protein kinase	3893	10	341
SVR1	Suppressor of variegation 1	2400	5	112
TIC62	Translocon at inner chloroplast membrane 62	1830	8	343
Z-ISO	15-cis-zeta-carotene isomerase	1395	2	112
ZDS1	Zeta-carotene desaturase 1	1189	7	​
ZEP	Zeaxanthin epoxidase	2465	10	255
petC	Cytochrome b6-f complex iron-sulfur subunit	2125	4	194
Carbon assimilation and distribution				
AAE1	Acyl-activating enzyme 1	2950	8	350
ACR11	ACT domain repeat protein 11	2184	15	245
ACS	Acetyl-CoA synthetase	1982	9	​
ACX2	Acyl-CoA oxidase 2	2501	8	441
AGD2	Aberrant growth and death 2	1590	9	442
AKN	Adenylate kinase, nuclear/plastid-type	1340	6	​
AMY2	Alpha-amylase 2	2836	8	321
APT3	Adenine phosphoribosyltransferase 3	1928	7	194
BAM8	Beta-amylase 8	2677	6	531
BCAT1	Branched-chain amino acid aminotransferase 1	2730	3	256
BCKDH-E1α	Branched-chain alpha-keto acid dehydrogenase E1	2428	14	263
D2HGDH	D-2-hydroxyglutarate dehydrogenase	2328	12	288
ENO	Enolase	1954	7	140
EPSPS-2	5-enolpyruvylshikimate-3-phosphate synthase 2	1554	6	​
FATB1	Acyl-ACP thioesterase FATB1	2446	9	410
FBPban1	Fructose-1,6-bisphosphatase	1666	17	191
FDH1	Formate dehydrogenase 1	2895	5	373
FPGS2	Folylpolyglutamate synthetase 2	2348	21	441
GAL1	Galactokinase 1	1044	13	​
GDCSH	Glycine decarboxylase complex H subunit	1663	4	​
GGL2	Gamma-glutamyl ligase 2	1949	9	218
GGT3	Gamma-glutamyl transferase 3	4165	7	395
GMPM1	GDP-mannose pyrophosphorylase 1	1402	3	202
HPR-A	Hydroxypyruvate reductase A	2428	7	333
IIL1	Isopropylmalate isomerase large subunit 1	4360	13	636
IPUT1	Inositol phosphorylceramide glucuronosyltransferase	2365	7	102
KAS3A	3-ketoacyl-ACP synthase 3A	1728	8	391
LACS7	Long-chain acyl-CoA synthetase 7	2539	14	701
LPAT2	Lysophosphatidic acid acyltransferase 2	2747	14	381
LPD1	Dihydrolipoamide dehydrogenase 1	2883	4	385
METE	Methionine synthase	822	5	190
MIOX1	Myo-inositol oxygenase 1	3088	11	230
MTN1	Methylthioadenosine nucleosidase 1	1301	10	275
PFK5	Phosphofructokinase 5	4147	15	386
PGDH3	3-phosphoglycerate dehydrogenase 3	2505	3	356
PGM1	Phosphoglucomutase 1	2280	10	380
PPOX2	Protoporphyrinogen oxidase 2	1028	13	154
PPT1	Phosphoenolpyruvate/phosphate translocator 1	1556	5	358
PTST	Protein targeting to starch 1	1662	7	298
PUR2	Phosphoribosyl formyl glycinamidine synthase 2	1219	9	406
RBCMT	Rubisco methyltransferase	2291	12	281
RBCS	Ribulose bisphosphate carboxylase small chain	303	1	​
SDH1-1	Succinate dehydrogenase 1-1	2862	9	545
SIR	Sulfite reductase	5547	10	526
SPS3	Sucrose-phosphate synthase 3	3839	9	885
TAT7	Tyrosine aminotransferase 7	4072	8	297
TPS4	Trehalose-6-phosphate synthase 4	1564	4	​
XPT	Xylulose 5-phosphate/phosphate translocator	1979	2	421
Antioxidant/Redox homeostasis				
AKR1	Aldo-keto reductase 1	2244	7	166
APX6	Ascorbate peroxidase 6	2886	7	130
BAG5	BCL2-associated athanogene 5	2338	4	334
CAT1	Catalase 1	2061	5	​
CBSX3	CBS domain-containing protein CBSX3	2257	6	206
DHAR3	Dehydroascorbate reductase	1926	7	​
GSTU17	Glutathione S-transferase	2434	3	119
NTRC	NADPH-dependent thioredoxin reductase C	1862	10	286
Pyroxd2	Pyridine nucleotide-disulfide oxidoreductase domain	3485	18	564
RCD1	Radical-induced cell death 1	4613	10	500
SABP2	Salicylic acid-binding protein 2	1840	5	113
SODCP	Superoxide dismutase, chloroplastic	1485	15	134
VTC4	GDP-L-galactose phosphorylase	604	1	107
Hormone signalling and crosstalk				
ABI1	ABA insensitive 1 (protein phosphatase 2C)	3403	17	562
APRR1	Arabidopsis pseudo-response regulator 1	3221	10	449
ARF18	Auxin response factor 18	3866	16	652
ARF4	Auxin response factor 4	781	5	260
BSK6	Brassinosteroid-signaling kinase 6	1824	8	268
CCA1	Circadian clock associated 1	2277	15	412
CYP707A2	Abscisic acid 8-hydroxylase CYP707A2	622	6	​
CYP734A1	Brassinosteroid-inactivating cytochrome P450 C	2239	6	375
CYP85A	Cytochrome P450 85A / brassinosteroid biosynthesis	3897	6	490
ERF008	Ethylene response factor 8	1007	5	172
GAF1	GAMYB-associated factor 1	2375	8	381
HAB1	Homolog of ABA-insensitive	3729	10	313
IBR3	Indole-3-butyric acid response 3	6334	15	407
JOX2	Jasmonate-induced oxygenase 2	876	3	​
LNK3	Night light-inducible and clock-regulated protein 3	1834	8	373
PIF3	Phytochrome-interacting factor 3	2621	8	346
PIF5	Phytochrome-interacting factor 5	4288	15	386
RVE1	REVEILLE 1	4135	12	218
RVE8	REVEILLE 8	1294	11	173
WNK8	With-no-lysine kinase 8	4174	6	523
ACA9	Autoinhibited Ca2+-ATPase 9	4267	16	1116
APY3	Apyrase 3	430	4	​
CIPK21	CBL-interacting protein kinase 21	1345	15	112
CNGC2	Cyclic nucleotide-gated ion channel 2	2770	4	552
CNGC20	Cyclic nucleotide-gated ion channel 20	3691	10	490
CPK12	Calcium-dependent protein kinase 12	981	10	162
CSC1	Calcium-permeable stress-gated cation channel 1	3267	11	610
HPCA1	Hydrogen peroxide-induced Ca2+ increases 1	3440	4	967
KIN4A	Kinase 4A	1062	3	227
LYK3	Lysin motif receptor kinase 3	2859	14	667
MICU	Mitochondrial calcium uptake regulator MICU	1954	10	146
MLO3	Mildew resistance locus O 3	3974	13	254
NIK1	NSP-interacting kinase 1	2687	11	622
OXI1	Oxidative signal-inducible 1 kinase	1589	2	267
PAD4	Phytoalexin deficient 4	2967	12	630
RBOHC	Respiratory burst oxidase homolog C	3530	5	884
REM4.1	Remorin 4.1	2309	6	634
RGI1	RGF insensitive 1 receptor kinase	1951	4	558
RGS1	Regulator of G-protein signaling 1	2774	9	465
ROPGEF14	Rho-of-plants guanine nucleotide exchange factor 14	2124	5	544
ROPGEF7	Rho-of-plants guanine nucleotide exchange factor 7	2452	5	705
RUS4	ROOT UV-B SENSITIVE 4	1313	10	​
TPC1	Two-pore channel 1	2786	5	613
VIP1	VirE2-interacting protein 1	4133	6	​
Transporters				
AAP7	Amino acid permease 7	1977	6	462
ADNT1	Adenine nucleotide transporter 1	564	2	114
AHA10	Plasma membrane H+-ATPase 10	2827	10	398
AHA11	Plasma membrane H+-ATPase 11	1203	5	​
ALA2	Aminophospholipid ATPase 2	4816	11	1030
CAX1	Cation/H+ exchanger 1	1760	15	419
CHC1	Clathrin heavy chain 1	1952	7	241
DTX16	Detoxification efflux carrier 16	1383	11	157
DTX17	Detoxification efflux carrier 17	1023	11	​
DTX35	Detoxification efflux carrier 35	2579	11	477
DTX40	Detoxification efflux carrier 40	1511	5	​
FRO6	Ferric reduction oxidase 6	674	5	​
KEA1	K+ efflux antiporter 1	2078	4	​
LTPG5	Lipid transfer protein GPI-anchored 5	948	2	189
OPT7	Oligopeptide transporter 7	3547	4	482
PDR1	Pleiotropic drug resistance 1	2496	4	341
PICALM1B	Phosphatidylinositol-binding clathrin assembly protein	1587	9	102
PTR1	Peptide transporter 1	2344	4	104
RABGAP22	Rab GTPase-activating protein 22	2692	6	548
SKOR	Stelar K+ outward rectifier	3219	7	728
SUC1	Sucrose transporter 1	2422	5	222
SWEET14	SWEET sugar transporter 14	1494	3	​
SYP71	Syntaxin of plants 71	2499	10	260
UMAMIT2	Amino Acid Transporter 2	1682	6	383
UMAMIT36	Amino Acid Transporter 36	2223	8	279
VHA-e1	Vacuolar H+-ATPase subunit e1	1824	12	​
VIT1	Vacuolar iron transporter 1	932	2	250
VTI13	Vesicle transport v-SNARE 13	1394	7	168
Transcription factors and regulators				
AHL14	AT-hook motif nuclear-localized protein 14	1533	4	336
ATHB-12	Arabidopsis thaliana homeobox protein 12	1105	3	230
BHLH089	Basic helix-loop-helix transcription factor 089	1224	12	220
BHLH130	Basic helix-loop-helix transcription factor 130	1863	6	219
BHLH48	Basic helix-loop-helix transcription factor 48	1907	13	364
BHLH63	Basic helix-loop-helix transcription factor 63	1629	3	264
BHLH68	Basic helix-loop-helix transcription factor 68	633	13	105
CBP20	Cap-binding protein 20	1566	6	267
DXO1	DXO1 decapping/exoribonuclease	3069	7	332
GATA16	GATA transcription factor 16	1113	3	​
MYB3R1	MYB3R1 transcription factor	2543	12	487
MYB3R3	MYB3R3 transcription factor	1716	2	425
NAC090	NAC domain-containing protein 90	1323	4	157
NCBP	Nuclear cap-binding protein	3222	9	​
NFYC4	Nuclear transcription factor Y subunit C-4	4029	5	203
NOF1	Nucleolar factor 1	2625	7	422
ORRM1	Organelle RNA recognition motif protein 1	2110	5	390
PABN3	Poly(A)-binding protein nuclear 3	1115	7	​
POL	POLTERGEIST phosphatase	569	10	​
RLUA1/FCS1	rRNA pseudouridine synthase RLUA1 / FCS1	2155	21	128
SR30	Serine/arginine-rich splicing factor 30	1020	13	112
U2AF35B	U2 small nuclear ribonucleoprotein auxiliary factor	2975	6	148
WRKY1	WRKY transcription factor 1	2065	2	​
WRKY14	WRKY transcription factor 14	2470	2	468
WRKY6	WRKY transcription factor 6	2375	4	530
BXL1	Beta-xylosidase 1	3282	7	771
FH5	Formin homology protein 5	4981	3	626
FIM2	Fimbrin 2	2500	3	667
ICR1	Interactor of constitutively active ROP 1	3087	11	376
IQD5	IQ-domain 5	2269	15	326
KIN14B	Kinesin-14B	1174	5	362
KUA1	KUA1 cell-expansion regulator	1670	4	230
VLN4	Villin 4	4087	9	963
WLIM1	LIM domain protein WLIM1	1404	5	100
XTH23	Xyloglucan endotransglucosylase/hydrolase	753	1	143

### 3.5 Validation of differential gene expression through real time PCR

To validate the RNA-seq results, we used quantitative real-time PCR (qRT-PCR) to investigate the expression patterns of eight representative differentially expressed genes (*CcABl1, CcENL, CcGSTU, CcLHCB5, CcPFK5, CcPGM1, CcRBCS,* and *CcRVE1*) under various treatment conditions. The overall results of the qRT-PCR were consistent with the RNA-seq data and were highly correlated with it (Pearson’s correlation coefficient, r = 0.60; *R* ^2^ = 0.36; p = 1.06 × 10^−6^) (Figure 8). The majority of the selected genes followed the same trend observed in RNA-seq data in response to various treatments indicating that the RNA-seq data was a good indicator of the expression patterns of the genes. Slight differences in expression levels were observed for a few gene–treatment combinations presumably due to the different normalization procedures used in RNA-seq during the library preparation.

**FIGURE 8:**
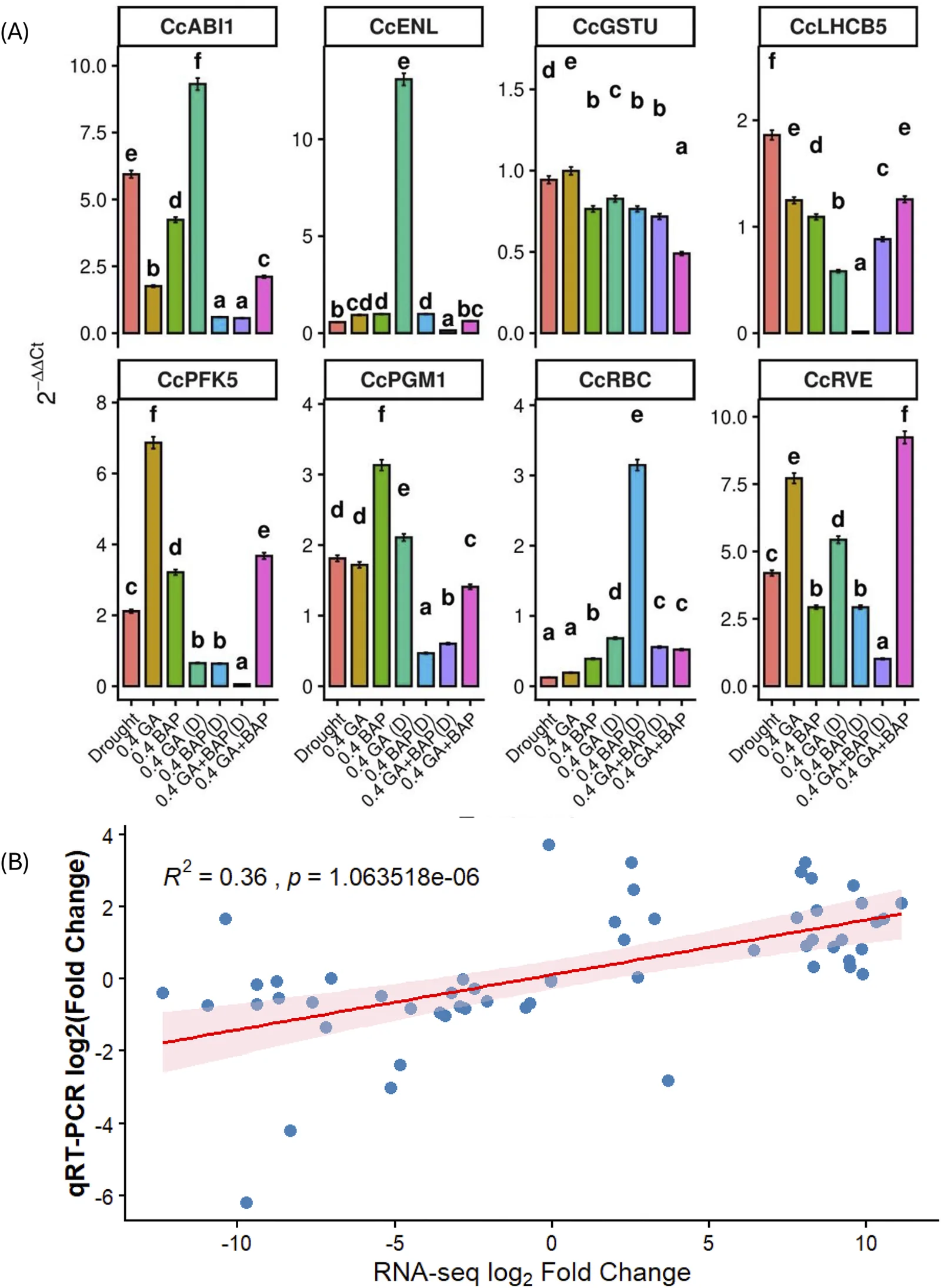
Validation of RNA-seq expression profiles by qRT-PCR and correlation analysis. **(A)** Relative expression levels (2^−ΔΔCT^) of targeted genes (*CcABI1, CcENL, CcGSTU, CcLHCB5, CcPFK5, CcPGM1, CcRBCS,* and *CcRVE*) across eight experimental groups. Values are presented as mean ± SD (n = 3). Data were analyzed using one-way ANOVA followed by Tukey’s HSD test. Means sharing one or more lowercase letters are not significantly different, whereas means with different lowercase letters differ significantly (*p* < 0.05). **(B)** Correlation between RNA-seq and qRT-PCR expression data for the eight selected genes across all treatment conditions. Each point represents a gene–treatment combination. The red line indicates the fitted linear regression, and the shaded area represents the 95% confidence interval. A significant positive correlation was observed between the two datasets (Pearson’s *r* = 0.60; *R* ^2^ = 0.36; *p* = 1.06 × 10^−6^).

### 3.6 Integrated gene–metabolite association network analysis

Integrated gene–metabolite association network analysis revealed strong associations between genes and metabolites under drought and hormone treatments (Figure 9). The network was constructed based on the significant Pearson correlations (|r| ≥ 0.80, P < 0.05) and visualised with Cytoscape (v3.10.4), and the topology of the network was analysed with the Network Analyzer tool. The association network comprised two major clusters with distinct association patterns. One major cluster contained highly connected hub genes, including *AMY2, METE, CAO, PNSB4, VLN4, KEA1, SR1IP1, ARF4 and CAX1*, which were associated with metabolites involved in flavanol, carotenoid and carbohydrate metabolism. Metabolites that were highly connected were limonexic acid, tetrahydrocurcumin and glucoside-containing compounds and were linked to stress-responsive genes. The second major cluster had a relatively modular structure and included hub genes such as *VIP1, RBCMT, TPS4, PLSB, VTC4, SN2, IDD4 and UTP6*, which were associated with metabolites including arbutin, pyridine derivatives and hexose phosphate-associated compounds. The network topology analysis identified a large number of connections among genes related to carbon metabolism, stress signaling, redox regulation and drought-responsive metabolites.

**FIGURE 9:**
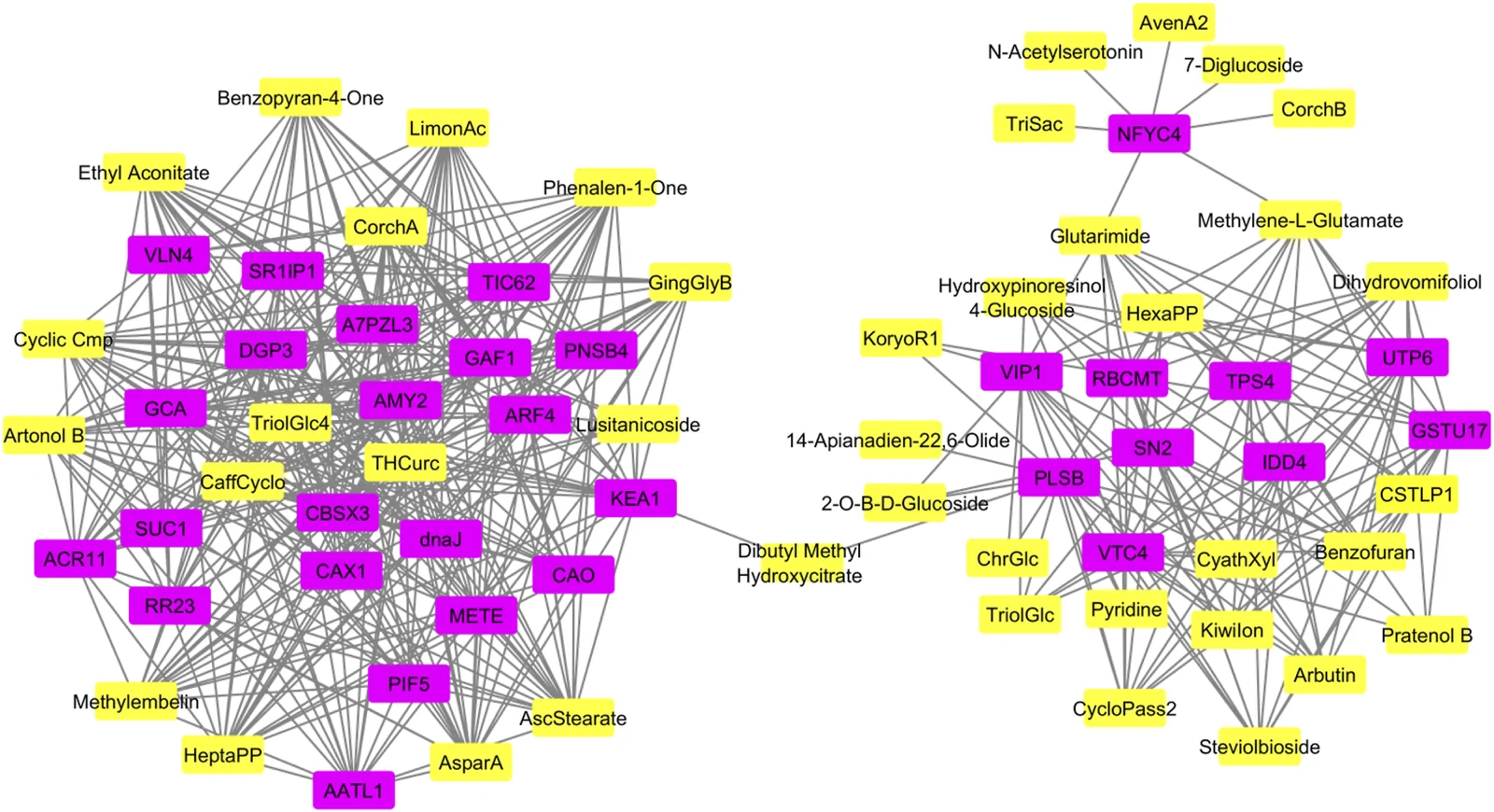
Gene–metabolite association network in *Cajanus cajan* under hormone treatments and drought stress. Network showing significant associations between differentially expressed genes (magenta nodes) and metabolites with varied abundance (yellow nodes) identified from the comparisons of control *versus* drought, control *versus* GA_3_, control *versus* 6-BAP, control *versus* GA_3_+6-BAP, and GA_3_ *versus* GA_3__D, 6-BAP *versus* 6-BAP_D, and GA_3_+6-BAP *versus* GA_3_+6-BAP_D. Edges represent significant Pearson correlations (|r| ≥ 0.80, P < 0.05). The right panel highlights key subnetworks and hub genes and metabolites identified based on degree centrality.

## 4 Discussion

### 4.1 Gibberellic acid and 6-benzylaminopurine sustain photochemical efficiency and thylakoid energy partitioning under drought stress

Drought stress critically impacts plant growth by interfering with water transport, carbon assimilation and metabolic homeostasis. Phytohormone signaling has emerged as an important regulator of plant adaptation to abiotic stress through coordinated modulation of morphophysiological and metabolic processes. In particular, the interplay among gibberellins, cytokinins and ABA regulates stress adaptation by influencing antioxidant defense, osmotic adjustment, and stomatal regulation (Shaffique et al., 2023). Despite considerable advances in understanding individual hormone functions, studies examining the coordinated effects of exogenous gibberellin and cytokinin on photosynthetic performance, thylakoid energy partitioning, and carbon metabolism under drought stress conditions remain limited, particularly in legumes. Similar to many legume and non-legume crop species drought stress in pigeonpea substantially reduced growth, leaf hydration, photosynthetic performance, and metabolic stability, consistent with impaired photosynthetic carbon assimilation and altered source–sink relationships under water-deficit conditions (Pinheiro and Chaves, 2011; Qiao et al., 2024; Sreeharsha et al., 2015). Endogenous bioactive GA_3_ levels generally decline during drought as part of an adaptive growth-stress tradeoff that restricts cell expansion and growth through DELLA-mediated hormonal regulation (Hedden and Thomas, 2012; Shani et al., 2024). Similarly, reduced cytokinin levels accelerate leaf senescence, impair cellular hydration, and diminish sink activity, whereas exogenous 6-BAP delays these responses presumably through interactions with ABA-mediated stress signaling pathways (Rivero et al., 2007). In the present study, exogenous GA_3_ and 6-BAP substantially alleviated the detrimental effects of drought on LRWC and biomass accumulation, indicating improved maintenance of cellular hydration and photosynthetic productivity under stress. Notably, GA_3_ alone produced greater improvements in vegetative biomass than 6-BAP, suggesting preferential allocation of assimilates towards vegetative growth. However, the combined application of GA_3_ + 6-BAP (0.4 mM) resulted in superior biomass and yield under both well-watered and drought conditions, which could be associated with improved source–sink coordination and more efficient carbon partitioning during drought acclimation.

Chlorophyll *a* fluorescence provides a sensitive indicator of PSII stability under environmental stress conditions to understand the photochemical efficiency and its regulation under drought stress (Sekhar et al., 2015). Both RLC and slow induction kinetics illustrated that drought impaired photochemical efficiency by reducing electron transport capacity and limiting the utilization of absorbed light energy, thereby restricting carbon assimilation (Flexas et al., 2004; Kalaji et al., 2016). The marked decline in ETR_max_ under drought indicates reduced electron transport capacity, thereby decreasing the capacity to support ATP synthesis and reducing power generation required for carbon fixation (Baker and Rosenqvist, 2004; Maxwell and Johnson, 2000). In contrast, hormone-treated plants, especially GA_3_ and GA_3_+6-BAP, maintained high ETR_max_ and Ek under drought stress implying better use of absorbed excitation energy and preservation of PSII photochemical activity. Increased ETR in hormone-treated plants was accompanied by improved biomass accumulation suggesting maintenance of photosynthetic productivity under stress. Furthermore, SP kinetics showing relatively stable steady-state PSII activity in hormone-treated plants indicate preservation of reaction center functionality under high irradiance, representing an adaptive response that helps prevent stress-induced photoinhibition. The fluorescence transients observed during the induction and relaxation phases suggest improved coordination between photosystem II and photosystem I and indicate enhanced adjustment of the photosynthetic apparatus to changing environmental conditions, supporting hormone-mediated stress tolerance in pigeonpea. Non-photochemical quenching plays a central role in balancing photochemical energy utilization with thermal dissipation of excess excitation energy during stress. The significant increase in NPQ observed in untreated drought-stressed plants reflects enhanced thermal dissipation under conditions of restricted carbon assimilation and elevated excitation pressure. In contrast, hormone-treated plants maintained comparatively lower NPQ together with higher Y(II) and ETR indicating more efficient utilization of absorbed light energy for photochemistry while reducing the requirement for excessive thermal dissipation. The decrease in NPQ in drought plants treated with GA_3_ and GA_3_+6-BAP was correlated with increased Y(II) and ETR, pointing to an increased use of absorbed light energy for photochemical electron transport and a decreased need to dissipate excess energy as heat. The greater NPQ under drought stress in the untreated plants is probably a photoprotective response to the elevated excitation pressure, which allows the dissipation of excess absorbed energy through heat. These responses suggest improved coordination between light harvesting, photochemical energy conversion, and photoprotective mechanisms in hormone-treated plants.

The proton motive force (pmf) measurements further demonstrated that drought altered thylakoid energy partitioning by affecting both the ΔpH and the ΔΨ components of the electrochemical proton gradient. Perturbation of these components comprises photosynthetic energy conversion, because ΔpH regulates ATP synthase activity and NPQ induction, whereas ΔΨ contributes to charge balance across the thylakoid membrane (Kanazawa et al., 2017). Hormone-treated plants exhibited comparatively stable pmf, ΔpH and ΔΨ under drought stress, indicating improved maintenance of thylakoid electrochemical energy balance and more efficient coupling between electron transport and ATP synthesis. Efficient photochemical performance and thylakoid energy balance is expected to influence downstream carbon metabolism. Hormone treatments were shown to regulate photosynthetic energy partitioning thus assisting in drought acclimation of pigeonpea.

### 4.2 Flavonoids, phenolics along with lipid-associated metabolites contribute to redox homeostasis under gibberellic acid and 6-benzylaminopurine treatment

The physiological observations were accompanied by marked biochemical changes associated with carbon and redox metabolism. Drought stress substantially decreased chlorophyll, carotenoid, carbohydrate, and protein contents together with alterations in the C/N and Hex/Suc ratios, which suggests a disruption of carbon–nitrogen coordination and sugar metabolism. The decline in photosynthetic pigments reflects destabilisation of the photosynthetic apparatus and increased susceptibility to photooxidative damage. In contrast, hormone-treated plants maintained higher chlorophyll and carotenoid contents, suggesting enhanced stability of photosynthetic membranes and oxidative balance under drought conditions. Likewise, the comparatively lower C/N ratio observed following the combined GA_3_+6-BAP treatment further highlights improved nitrogen assimilation and metabolic coordination during stress adaptation. Changes in Hex/Suc ratio further indicate altered sucrose turnover and increased hexose availability, which may support osmotic adjustment and respiratory metabolism during drought. Similar metabolic flexibility has been associated with improved drought adaptation in several crop species (Fàbregas and Fernie, 2019). These biochemical responses are consistent with the higher expression of carbohydrate metabolism-related genes, including *AMY2, PGM1* and *ENO* which participate in starch degradation, glycolysis, and downstream carbon metabolism, thereby supporting metabolic adjustment under drought stress.

Drought markedly reduced the abundance carbohydrate-, lipid- and organoheterocyclic-associated metabolites, reflecting disruption of primary metabolism, osmotic regulation, and membrane-associated metabolic processes. In contrast, the combined GA_3_+6-BAP treatment partially restored these metabolite classes and shifted the overall metabolic profile away from the drought-associated state, as evidenced by the PCA analysis. Comparable hormone-mediated metabolic adjustments under water deficit have been reported in maize and soybean (Wang Y. et al., 2023; Zhao et al., 2025) suggesting that coordinated metabolic reprogramming represents a conserved response to drought stress in crop plants. Hormone treatments also improved metabolites involved in osmotic adjustment and stress adaptation, including carbohydrate-related compounds such as glucocaffeic acid together with glucoside-containing compounds, as well as phenylpropanoid- and defense-related metabolites, including 3-O-p-coumaroylquinic acid. The increased abundance of these metabolites coincided with improved LRWC, greater carbohydrate accumulation, and enhanced photochemical efficiency, suggesting coordinated regulation of carbon metabolism and osmotic homeostasis during drought acclimation.

The data further demonstrated substantial enrichment of flavonoid and phenolic metabolites following hormone treatments. Increased accumulation of peonidin derivatives, quercetin conjugates, and epicatechin-related metabolites suggests enhanced antioxidant capacity and improved regulation of cellular redox homeostasis. Flavonoids and phenolics constitute important non-enzymatic antioxidants that scavenge ROS and protect the photosynthetic structures and cellular membranes under environmental stress (Agati et al., 2012; Sharma et al., 2023). Likewise, the recovery of lipid-associated metabolites following GA_3_ and GA_3_+6-BAP treatments indicates improved membrane integrity and stress-responsive lipid metabolism during drought stress. Such lipid remodelling is likely to contribute to membrane stability and sustained photosynthetic performance under water-deficit conditions. Among the secondary metabolites, terpenoid-associated compounds also exhibited pronounced treatment-dependent responses. The increased abundance of metabolites such as cyclopassifloside II following 6-BAP and GA_3_+6-BAP treatment and limonexic acid following GA_3_+6-BAP application suggests activation of terpenoid metabolism during drought acclimation. Terpenoids have been widely implicated in stress signalling, membrane protection, and antioxidant defence during abiotic stress, and their enrichment implies activation of protective secondary metabolic pathways under hormone treatment. Although untargeted LC-HRMS analysis provided broad coverage of drought- and hormone-responsive metabolites, most metabolite signatures were assigned through accurate mass matching and putative database annotations. Therefore, these metabolic features may require further confirmation using targeted validation under drought and hormonal interaction.

### 4.3 Transcriptional networks of carbon assimilation and antioxidative metabolism were in coordination with physiological and metabolic adaptations

The physiological and metabolic adjustments induced by hormone treatments were accompanied by extensive transcriptional reprogramming, as indicated by transcriptome profiling. Drought stress suppressed the expression of several genes associated with photosynthesis and carbon metabolism including *RBCS, PPD5, STN7* and *ENO*, consistent with the decline in PSII efficiency and carbon assimilation observed under water-deficit conditions. In contrast, the combined GA_3_+6-BAP treatment maintained comparatively higher expression of genes involved in carbon assimilation and glycolysis, including *FBPBan1* and *ENO*, suggesting preservation of carbon metabolism and downstream metabolic flux during drought stress. Hormone treatments also maintained higher transcript abundance of photosynthetic regulatory genes, including *PSBS* and *STN7*, indicating improved regulation of light harvesting and photochemical processes. PSBS is a central component of photoprotective energy dissipation, and its increased expression in hormone-treated plants likely contributed to balancing photochemical regulation during drought rather than acting as an independent determinant of drought tolerance. Consistent with this interpretation, the higher expression of PSBS occurred together with improved chlorophyll fluorescence parameters indicating coordinated regulation of photoprotection and photochemical performance under hormone treatment. Hormone-mediated drought acclimation was further associated with coordinated regulation of redox homeostasis and stress signaling pathways. The higher transcript abundance of *APX6, CAT1* together with ROS-signalling gene *RBOHC* suggests simultaneous activation of antioxidant defense and redox signaling during drought adaptation. Likewise, enhanced expression of calcium-signaling genes including *CNGC2, CRK3, CRK12* and *ACA9* is associated with activation of calcium-mediated and receptor-like kinase signaling pathways that are widely recognized as central regulators of abiotic stress responses (Wang T. et al., 2023). The consistency between RNA-seq and qRT-PCR analyses further validated the transcriptional responses observed in the present study. Integration of transcriptomic and metabolomic datasets provided additional insight into the coordinated regulation of drought-responsive pathways under hormone treatments. The gene–metabolite association network revealed strong relationships between genes involved in carbon metabolism, redox regulation, and signalling pathways and metabolites associated with carbohydrate turnover, flavonoid biosynthesis, lipid metabolism, and other secondary metabolic processes. These coordinated associations support the concept that transcriptional regulation and metabolic reprogramming operate together to maintain carbon metabolism, redox homeostasis, and photosynthetic function during drought stress. The present transcriptomic dataset provides a comprehensive overview of hormone-responsive transcriptional reprogramming under drought stress. However, the identified differentially expressed genes should primarily be regarded as candidate regulators of hormone-mediated drought responses. Future studies involving resequencing, together with functional characterization of key hub genes identified in the integrated gene–metabolite network, will be essential to validate these regulatory pathways and elucidate their precise roles in drought adaptation. Collectively, these findings indicate that coordinated regulation of photosynthetic efficiency, thylakoid energy partitioning, and stress-responsive transcriptional networks underpins the enhanced drought tolerance observed following hormone treatment, particularly 0.4 mM GA_3_ + 6-BAP application. Furthermore, the candidate hub genes identified through degree-centrality analysis of the integrated gene–metabolite association network represent promising targets for future functional characterization and molecular breeding of drought-resilient legume crops.

## 5 Conclusion

Drought stress significantly reduced growth, photosynthetic efficiency, and metabolic stability in pigeonpea. Exogenous application of integrated GA_3_ and 6-BAP formulation (0.4 mM) mitigated drought-induced impairment and improved carbon assimilation, photochemical efficacy, energy distribution thereby enhancing the overall plant performance under stress. The metabolomic analysis showed a significant increase in flavonoids, phenolics, and terpenoid compounds upon hormonal treatments which are likely associated with improved protection against drought stress. In addition, transcriptome analysis showed that there was coordinated changes in transcript abundance of genes associated with photosynthesis, redox regulation and calcium-mediated signalling, indicating coordinated stress adaptations in response to drought conditions. In conclusion, the results offer insights into the mechanism of drought tolerance in pigeonpea and indicate that the combination of 0.4 mM GA_3_ and 6-BAP could enhance plant performance under water-limited conditions.

## Data Availability

The original contributions presented in the study are publicly available. This data can be found in the NCBI repository with the BioProject accession number PRJNA1457079.
